# Cost effectiveness analysis comparing repetitive transcranial magnetic stimulation to antidepressant medications after a first treatment failure for major depressive disorder in newly diagnosed patients – A lifetime analysis

**DOI:** 10.1371/journal.pone.0186950

**Published:** 2017-10-26

**Authors:** Jeffrey Voigt, Linda Carpenter, Andrew Leuchter

**Affiliations:** 1 Medical Device Consultants of Ridgewood, LLC, Ridgewood, NJ, United States of America; 2 Department of Psychiatry and Human Behavior, Brown Institute for Brain Science, Brown University, Providence, RI, United States of America; 3 Neuromodulation Division, Semel Institute for Neuroscience and Human Behavior, University California Los Angeles, Los Angeles, CA, United States of America; Nanjing Normal University, CHINA

## Abstract

**Objective:**

Repetitive Transcranial Magnetic Stimulation (rTMS) commonly is used for the treatment of Major Depressive Disorder (MDD) after patients have failed to benefit from trials of multiple antidepressant medications. No analysis to date has examined the cost-effectiveness of rTMS used earlier in the course of treatment and over a patients’ lifetime.

**Methods:**

We used lifetime Markov simulation modeling to compare the direct costs and quality adjusted life years (QALYs) of rTMS and medication therapy in patients with newly diagnosed MDD (ages 20–59) who had failed to benefit from one pharmacotherapy trial. Patients’ life expectancies, rates of response and remission, and quality of life outcomes were derived from the literature, and treatment costs were based upon published Medicare reimbursement data. Baseline costs, aggregate per year quality of life assessments (QALYs), Monte Carlo simulation, tornado analysis, assessment of dominance, and one way sensitivity analysis were also performed. The discount rate applied was 3%.

**Results:**

Lifetime direct treatment costs, and QALYs identified rTMS as the dominant therapy compared to antidepressant medications (i.e., lower costs with better outcomes) in all age ranges, with costs/improved QALYs ranging from $2,952/0.32 (older patients) to $11,140/0.43 (younger patients). One-way sensitivity analysis demonstrated that the model was most sensitive to the input variables of cost per rTMS session, monthly prescription drug cost, and the number of rTMS sessions per year.

**Conclusion:**

rTMS was identified as the dominant therapy compared to antidepressant medication trials over the life of the patient across the lifespan of adults with MDD, given current costs of treatment. These models support the use of rTMS after a single failed antidepressant medication trial versus further attempts at medication treatment in adults with MDD.

## Introduction

Repetitive transcranial magnetic stimulation (rTMS) is a non-invasive method of stimulating human brain tissue using strong, time varying magnetic fields to induce small currents in the nerve tissue. Companies available in the US that have received Food and Drug Administration (FDA) clearance for rTMS use in major depressive disorders (MDD) include: Magstim [1], Brainsway [2], Magventure [3], Neuronetics [4], and Neurosoft [5], with additional companies likely to soon follow. The therapy generally consists of 36 treatment sessions performed five times per week over a six-week period followed by a three-week taper phase. The treatment also may be beneficial for periodic follow on rTMS therapy depending upon the condition of the patient [6]. All of the rTMS products are indicated for: *“Treatment of major depressive disorder episodes in adult patients who have failed to receive satisfactory improvement from prior antidepressant medication in the current episode* [2–5].*”* This infers failure from at least one prior antidepressant medication trial.

A number of randomized controlled trials, systematic reviews, and meta-analyses (of randomized controlled trials) demonstrate the clinical efficacy of rTMS in the relief and improvement of MDD [7]. Several cost-effectiveness analyses have compared rTMS with pharmacologic therapies [8,9] and electroconvulsive therapy (ECT) [10–12]. One limitation of these cost effectiveness analyses is that they evaluate rTMS over short periods of time–e.g. nine weeks [9], one year [10–12], and, three years [8]–rather than the lifespan of a patient. Additionally, these analyses have examined the cost effectiveness of rTMS after several (more than one) failed antidepressant medication trials [8]. As a result, it is not known whether rTMS would be a cost effective therapy over the longer term (i.e. life of the patient) earlier in the treatment trajectory i.e., after a single failed medication trial.

The issue of how early in the course of illness rTMS should be introduced is particularly important. The likelihood of achieving remission with medication diminishes with each successive failure of the pharmacologic therapy [13–16]. Additionally, the FDA “Indications for Use” label for rTMS therapeutic devices suggests rTMS be used after as few as a single failed medication trial [17–20]. While clinical recommendations call for the use of rTMS after a patient has received 1–4 adequate antidepressant medication attempts [6], nearly all current major payer coverage policies (Medicare and private insurance) call for rTMS to be used after a patient with MDD is deemed refractory to other therapies over numerous trials. The coverage policies from the largest US payers commonly call for: at least four trials of psychopharmacologic agents (including at two different agent classes), a course of psychotherapy, and/or that the patient is a candidate for and has declined ECT [21–25]. In total, the four largest US private payer health plans cover >50% of the approximately 165 million total private payer enrollees, with Medicare covering approximately 45 million lives of the ≥65 years of age population. As a result of these policy guidelines, rTMS generally is performed primarily on patients shown to be highly refractory to other therapies and may therefore have a lower likelihood of success [13].

Data that could be used to demonstrate the cost-effectiveness of introducing rTMS as a treatment earlier in the course of depressive illness is extremely limited. As an alternative, Markov modeling can be used to evaluate the effect of earlier rTMS therapy and its cost-effectiveness over the life of a patient. Markov modeling commonly is used to simulate both the short-term (e.g., acute response to rTMS) and long-term (e.g., lifetime) value a therapy to the medical community and patients. These models use initial probabilities of response rates to therapies (based upon high quality published studies such as randomized controlled trials) and transition probabilities (e.g. likelihood of sustaining a health state of remission during recurrent treatment or maintenance therapy) after an initial treatment course. Lastly, Markov models also can be used to compare the direct costs of each of the treatments employed and the care for the health transition state over long periods of the patients’ lifetime.

We report herein the results of Markov models developed to evaluate the cost effectiveness of rTMS over the lifetime of the patient. These models are used to compare the cost-effectiveness of rTMS versus antidepressant drug therapy after a single failed antidepressant medication trial in newly diagnosed MDD patients and; over the course of their adult lifespan (in the age cohorts of mid 20’s, 30’s, 40’s and 50’s).

## Methods

A health transition state (Markov) decision tree model was used to evaluate the direct healthcare costs and outcomes (quality of life) of rTMS versus antidepressant therapy (two separate arms of a decision tree) over the lifetime of the patient. This model compared long term outcome from time of diagnosis through the end of expected life [26,27] using either rTMS or antidepressant medication after a first failed medication trial. The main outcomes were the direct costs of care and quality of life (as measured by Euro-Quality of Life (QoL), a standardized and validated instrument for use as a measure of health outcome). Euro- QoL is applicable to a wide range of health conditions and treatments, providing a single index value for the health status of a patient on a scale from 0–1: “1” indicates a state of perfect health and “0” indicates death. This model was used to compare rTMS to antidepressant drug therapy on the outcomes of cost and quality of life. A therapy that is more cost-effective (that is, costs less and improves quality of life) is said to dominate another therapy as it relates to cost-effectiveness.

### Model structure

A Markov simulation model for the entire life of the patient was constructed using TreeAge Pro 2017 software. The model assumed that the patients were in the age cohorts of mid 20’s, 30’s, 40’s and 50’s at time of diagnosis with MDD. rTMS was the next therapeutic intervention after a single failed medication trial. The life expectancy of patients with MDD was included in the model based upon available data (Table 1). Various health states were used based on treatments and as well, clinical outcome (remission, responder, relapse, nonresponse, maintenance, death). Patients entered the model and transitioned to the various health states based on the probability of their responsiveness to their treatment.

**Table 1:** Expected additional years of life of patients with MDD in the age cohorts evaluated in the model.

Age cohort	Expected additional years of life	Reference
Mid 20’s	Likely: 48; (Range: 33–63)	[26]
Mid 30’s	Likely: 38 (Range: 23–53)	[26]
Mid 40’s	Likely: 28 (Range 13–43)	[26]
Mid 50’s	Likely: 16 (Range: 3–29)	[27]

If a patient did not respond to either therapy or relapsed after remission, the model assumed that a second, third and fourth attempt was made to achieve remission with the same therapy (based on the probability of success/failure with that treatment). After a fourth non-response to either rTMS or antidepressant therapy, ECT was employed. Those who responded or remitted with a particular treatment were maintained on that therapy for the remainder of their life along with concurrent psychotherapy and maintenance antidepressant medications [28]. The model also accounted for patients entering and leaving states of remission and response, as well as not achieving response or remission with any therapy, based upon probabilities from the literature. Following nonresponse, the model assumed patients cycled through subsequent treatment options until one worked; if none produced a response the model assumed continued trials over time.

As an example, a patient could be treated with rTMS for 25.3±16.7sessions [29] over the course of a year and the treatment billed to an insurance company using Current Procedural Terminology (CPT) codes (90867 [initial treatment including motor threshold determination]; 90868 [subsequent treatments] and 90869 [subsequent motor threshold determination]) plus psychotherapy. The patient may respond (or not) to rTMS plus ongoing psychotherapy: if not responsive, the patient could be either retreated with rTMS or ultimately treated with another therapy (e.g. ECT). The patient then may either remit (assume further maintenance treatment with antidepressant medications plus psychotherapy) or respond (again requiring further treatment, e.g. additional rTMS plus antidepressants and psychotherapy). Lastly, a previous rTMS responder or remitter could relapse, in which case he or she would be retreated (with rTMS). For each of these states, the model calculated the associated costs for all modalities of concurrent treatment, including rTMS + antidepressant medications ± psychotherapy, based upon the likelihood of the particular health outcome, with probability of occurrence derived from the peer reviewed literature and displayed in S1 Appendix [variables] and/or S2 Appendix [distributions].

The “benefit” to the patient of each health outcome was modeled using Euro-QoL, with effects evaluated over the course of a patient’s treatments and resultant health states over their lifetime (see Health States below).

### Probabilities

The probabilities of treatment effects are listed in S1 and S2 Appendices were sourced from the peer reviewed published literature. Similarly, the treatment effect/success (of rTMS or pharmacotherapy) was based on the “diminished” probability of success after one failed pharmacotherapy trial as reported in the literature [8,16].

### Resources/treatments and costs

The direct costs of each treatment were based upon published 2016 national average Medicare reimbursement rates for rTMS procedures; for medications used to treat MDD; and for the 2016 Medicare reimbursement rates for psychotherapy [30]. Treatment sessions, duration of treatment, maintenance therapies, and periodic physician evaluations/management, were derived from the literature (S1 and S2 Appendices). The subsequent therapy for patients who were ultimately non-responders to either rTMS or numerous medication trials was ECT, which again was costed out using 2016 Medicare rates. Additional direct medical costs related to either rTMS or pharmacotherapy (e.g. inpatient, outpatient and emergency department) were derived from the literature [31] (S1 and S2 Appendices). Costs were discounted at 3% per year [32].

### Health states

Health states using the Euro-QoL visual analog scale, were derived from the literature and were assigned to each respective health state: baseline (depressed; diagnosed with MDD), response, non-response, remission, relapse (requiring re-treatment) and death. QoL assessments were modeled yearly and aggregated for the entire life of the patient. The aggregated number over the life of the patient is termed the quality adjusted life year (QALYs). A QALY is a generic measure of the burden of disease, and includes both the quality and the quantity of life lived. One QALY equals one year of life of perfect health (i.e. score of “1” on Euro-QoL). QALYs were discounted at 3% per year [32].

### Running the model and outcomes derived

**Fig 1:**
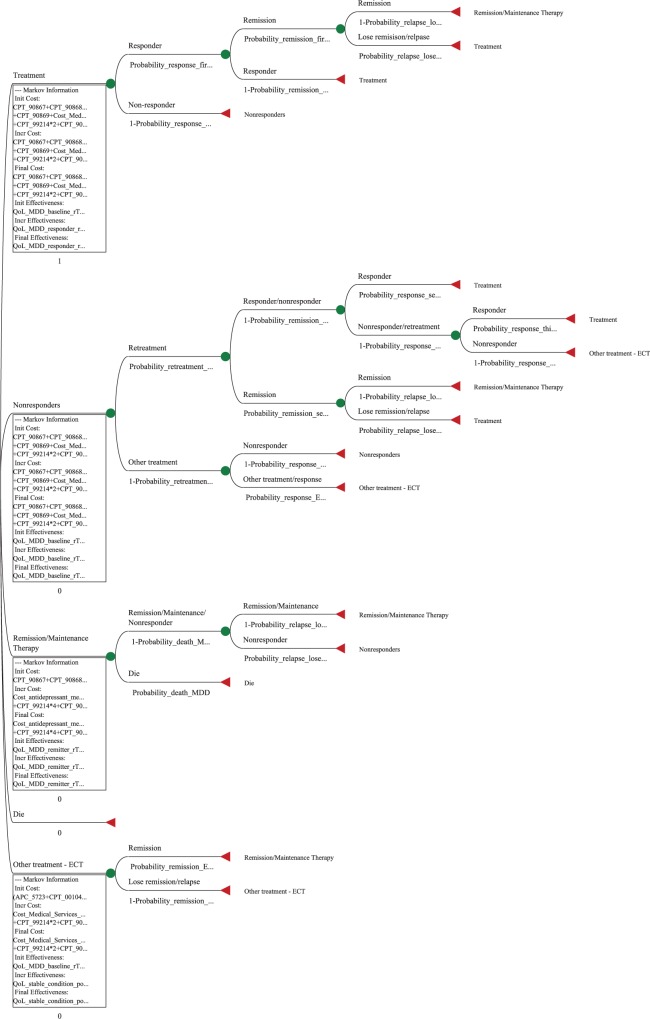
rTMS Markov model.

The main outcomes of the model were the aggregate direct costs and health states of patients as they transitioned through various health states over their entire remaining lifetimes. Baseline values were calculated. Monte Carlo simulation was run 1,000 times to determine the stability and consistency of the base line case using an incremental cost effectiveness scatterplot. A tornado diagram (diagram of a set of one-way sensitivity analyses compiled in one graph) was used to identify those variables that had an effect on choosing one therapy versus another based on the extent of change of the variable. Singular one-way sensitivity analysis was run on those variables which were determined to have the greatest effect on the model (i.e., the threshold value at which one would decide to use one treatment alternative over another based on being more cost-effective). Lastly, a cost-effectiveness graph was constructed to identify dominance of one therapy over the other. A portion of the Markov model is shown in Fig 1 depicting the rTMS therapy arm of the decision tree.

Net monetary benefits (NMB) were also calculated as a way to examine cost-effectiveness, with the most cost effective therapy having the highest NMB. The NMB is calculated as Effectiveness (in QALYS) multiplied by the “Willingness to Pay” less Costs. For this analysis, the willingness to pay was set to zero (0) so that the NMB value was negative. The NMB was evaluated for both arms of the decision tree. Since this NMB calculation resulted in a negative number for both arms of the decision tree, the least negative number (or the least costly therapy over the life of the patient) translated into the highest NMB. NMBs were expressed in both the tornado diagram and in the sensitivity analyses shown in the appendices.

Outputs to the model (by cost and QALYs per state and stage) were also calculated. The health states and their associated probabilities for each stage were “in treatment” (rTMS or pharmacotherapy), non-responder (undergoing follow on therapy), remission with maintenance therapy, in other treatment (i.e. ECT), and death. Each stage referenced a year of life. Definitions/terms used in the models are listed in S3 Appendix.

## Results

Application of the model at the start of the depression treatment trajectory demonstrated that rTMS was the dominant therapy as compared to pharmacotherapy (S1 Fig [Supplemental data]: Cost-Effectiveness Analysis; which demonstrates “dominance” of cost and QALY’s in the age 20’s cohort. Note: This dominance persisted in the age 30’s-50’s cohorts), costing less over the entire life of the patient and associated with better outcomes as measured by aggregated QoL scores or QALYs. The lifetime costs of treatment and QALYs by age cohort for rTMS, compared to pharmacotherapy, were lower for costs and higher for QALYs respectively (Table 2). Monte Carlo simulation of 1,000 trials of incremental cost effectiveness showed greater cost savings and improved QALYs with rTMS than antidepressant medications 36% to 40% of the time (S2–S5 Figs: Incremental Cost Effectiveness scatterplot, rTMS vs. antidepressant therapy [Supplemental data]). A tornado plot (S6–S9 Figs: Tornado Analysis [Net Monetary Benefits]; Supplemental data) shows which variables affected the model the most. These variables along with their threshold values (the value in which the other alternative becomes the more cost effective therapy or the highest NMB/least costly) can be seen in Table 3: Sensitivity Analysis.

**Table 2:** Expected values based on age cohort over the life of the patient.

Age cohort	rTMS (life time costs/lifetime QALYs)	Pharmacotherapy (life time costs/lifetime QALYs)
Mid 20’s	$278,103/15.22	$289,243/14.79
Mild 30’s	$257,686/14.06	$266,665/13.62
Mid 40’s	$226,126/12.26	$232,518/11.83
Mid 50’s	$164,769/8.77	$167,721/8.45

**Table 3:** One way sensitivity analysis–threshold values at which rTMS becomes the less costly alternative (S10–S21 Figs available in supplemental data section).

Variables	Values at which rTMS becomes the less costly alternative			
Age cohorts = = = = →	Mid 20’s	Mid 30’s	Mid 40’s	Mid 50’s
CPT 90868 (repeated rTMS)	<$273 (S10 Fig)	<$263 (S13 Fig)	<$250 (S16 Fig)	<$230 (S19 Fig)
Cost antidepressant therapy (monthly prescription cost)	>$276 (S11 Fig)	>$289 (S14 Fig)	>$306 (S17 Fig)	>$334 (S20 Fig)
Number rTMS sessions per year	<34 (S12 Fig)	<32 (S15 Fig)	<31 (S18 Fig)	<28 (S21 Fig)

The tornado plots indicate that the model is most sensitive to specific input variable assumptions. For patients who have failed one medication therapy, rTMS is a less costly therapy when: the number of rTMS sessions per year is less than 28 in the oldest [mid 50s age] and 34 in the youngest [mid 20s age] cohorts; the cost of rTMS/session or treatment is under $230 in the oldest [mid 50s age] and $273 in the youngest [mid 20s age] cohorts and; the cost of a one month supply of antidepressants medications is greater than $276 in the youngest [mid 20s age] and greater than $334 in the oldest [mid 50s age] cohorts. Interpreted differently, under reasonable circumstances (e.g. every day practice), rTMS is the less costly treatment alternative providing greater overall treatment efficacy over the life of the patient.

A separate (but related) question concerns the cost-effectiveness of rTMS treatment. Using the mid 50s age cohort as the example, the model evaluates how patients “fared” (cost of treatment and QALYs for each stage) in “cycling” through their therapeutic regimens, depending upon the treatment state they are in (treatment, non-responders, remission/maintenance therapy, other treatment [ECT] or death). rTMS and antidepressant drug therapy outcomes appear in S4 and S5 Appendices, respectively. Since there is a limited likelihood of patients entering remission with continued cycles of unsuccessful medication treatment, these appendices (S4 & S5) show each of the stages using the mid 50’s cohort as an example, listing a total of 16 different health stages, based on a patient living anywhere from 3–29 years, with 16 being the midpoint of the patient’s longevity and health states for each stage. The stages are listed along with their associated probabilities, costs, and QALYs. Additionally, each stage’s costs and QALYs are aggregated over time for overall cost and effectiveness. S6 Appendix describes the equations used for deriving the values appearing in Appendices S4 & S5. These data indicate that even if the cost of rTMS treatment were greater than the cost of medication in a given year, rTMS still provides for an improved quality of life (vs. medication) over the life of the patient (as measured in QALYs).

## Discussion

These analyses demonstrate that in patients newly diagnosed with MDD, rTMS can be a more cost-savings (having a higher NMB) /clinically effective therapy than further medication trials after a single failed antidepressant drug treatment—when considered over the entire life of the patient. In other words, rTMS had lower costs and overall higher QALYs over a patient’s lifetime when compared with multiple subsequent attempts of pharmacologic therapy after a single drug failure (S1 Fig [Supplemental data]). The pharmacy costs per month for prescription medications for treating MDD in newly diagnosed medication adherent patients was estimated @ $372.50/month [33]. These findings are consistent with previous shorter-term studies which demonstrate similar findings of dominance of rTMS versus pharmacotherapies [8]. The present findings extend this earlier work, demonstrating lower cost (a higher NMB)–improved effectiveness over the lifetime of the patient earlier in the disease treatment process. Use of rTMS earlier in the treatment process is consistent with the FDA labeling of the rTMS devices [17–20] and with studies of rTMS clinical efficacy [34,35]. The models presented here also incorporate a more refined probability of successful clinical outcome (i.e., the probability of depressive episode remission after one failed therapy) than other cost-effectiveness trials [10,11] that only examined patients with >1 and up to 4 failed pharmacotherapy trial as; these trials neglected to adequately address the probability of success based on the number of prior attempted treatment trials.

The only other cost-effectiveness analyses which addressed the probability of success based on the number of prior failed therapies (i.e. treatment resistance) were Nguyen et al [8]) and Simpson et al [9]. Nguyen et al. [8] demonstrated lower costs and higher QALYs (i.e. dominance or a higher NMB) of rTMS after at least 2 failed pharmacologic treatment regimens. In the present analysis, we have a finding of dominance (lower cost and higher QALYs) of rTMS after only one failed pharmacologic therapy. In Simpson et al [9], the comparator group was sham rTMS treatment, with a finding of a higher cost for active rTMS but higher QALYs.

The models presented herein also extend prior work by examining the lifetime effects of treating different aged cohorts of patients on either therapy, in contrast to earlier studies examined non-age specific patients [8] over a three-year timeframe. Other large sample durability studies only evaluate rTMS effects over at most one year [36].

The sensitivity analysis (tornado plot and values identified in Table 3) suggests that the present findings are likely robust (i.e., favoring rTMS as the preferred therapy and replicable in real life). The validity of these assumptions is supported by the literature. A meta-analysis of randomized, double blind, trials [7] demonstrated that patients derived clinically meaningful efficacy from an average of 12.6±3.9 rTMS sessions per year. The present analysis demonstrated that 28–34 treatments rTMS sessions/year could be performed (depending upon the age cohort) with rTMS still being more cost-effective. It is also likely that rTMS would have a clinically meaningful response under the 28-session threshold. Medicare reimburses at a national average rate of $206 per rTMS session (CPT 90868). The analysis also demonstrated that the CPT code reimbursement rate would have to exceed $230/rTMS session in order for rTMS to be less cost effective than pharmacologic therapy. Because the Medicare CPT code payment rate of $206 is less than the $230 threshold value, it is likely that rTMS would be the cost-effective therapy in practice. Lastly, in order for pharmacologic therapy to be more cost effective, the cost of a one month medication supply would need to be less than $276. Because the estimated cost of a one month supply of antidepressant medications in the model was $372 [33], exceeding a threshold cost of $276 would be unlikely to happen often in practice.

While it is possible that treatment with rTMS earlier in the trajectory of depressive illness might require fewer sessions than 34, data are currently not available to predict the number of sessions individual patients require to achieve optimal outcome.

### Incremental Cost Effectiveness Ratio (ICER) analysis–further analysis and interpretation of the data

If one examines the highest number of rTMS sessions used in the model in order to gain a therapeutic response (25.3±16.7 or a range from 8 to43 sessions), rTMS becomes a more costly therapy (having a lower NMB) than medication, with the ICER varying from $29,895 (mid 20’s) to $45,747 (mid 50’s) (Table 4). Therefore, even at the extreme end of the range of number of treatment sessions needed in order to obtain a response, rTMS would be still deemed cost effective based on US ICER thresholds of <$100,000/QALY [37]. In other words, since the cost/QALY is $29,895, and this number is lower than the US ICER threshold of $100K/QALY, it would be deemed to be cost effective per ICER standards of cost-effectiveness and; would likely be considered for incorporation into insurance coverage policies based on how insurers have perceived ICER analysis in the past.

**Table 4:** Incremental Cost Effectiveness Ratios [ICER] (Incremental cost rTMS vs. pharmacotherapy divided by the incremental benefit rTMS vs. pharmacotherapy)–assuming upper end of the number of treatments in order to have a response to rTMS and; assuming the cost of pharmacotherapy @ ~$100/month.

Age Cohort	Total cost rTMS	Total QALYs rTMS	Total cost pharma	Total QALYs pharma	ICER (Cost/QALY)
Number of rTMS sessions per month @ 43					
20’s	$302,098	15.22	$289,243	14.79	$12,855/0.43 = $29,895
30’s	$280,527	14.06	$266,665	13.62	$13,862/0.44 = $31,505
40’s	$247,184	12.26	$232,518	11.83	$14,666/0.43 = $34,107
50’s	$182,360	8.77	$167,721	8.45	$14,639/0.32 = $45,747
Cost of pharmacotherapy @ ~$100/month					
20’s	$237,049	15.22	$216,757	14.79	$20,293/0.43 = $47,193
30’s	$220,266	14.06	$199,838	13.62	$20,428/0.44 = $46,427
40’s	$194,325	12.26	$174,248	11.83	$20,077/0.43 = $46,691
50’s	$143,891	8.77	$125,690	8.45	$18,200/0.32 = $56,875

At the very low end of the range of pharmacotherapy costs per month (around $100/month for generic medications), again rTMS would become the costlier therapy with the ICERs varying from $47,193 (mid 20’s) to $56,875 (mid 50’s) (Table 4). In this circumstance, the ICER is again still <$100,000/QALY threshold in the 20’s–50’s cohorts. Interpreted differently, in order for a person to remain in perfect health for one year (i.e. QALY = 1) it would cost an additional $29,895-$56,875 using rTMS vs. pharmacotherapy; which per the US ICER threshold of <$100K/QALY would again be deemed cost effective per the threshold insurers use for determining cost-effectiveness. Even under the above conditions, rTMS maintains a cost effectiveness that payers have traditionally been willing to accept when considering this ICER threshold value for other therapies [38–42].

The use of rTMS in pharmacoresistant patients (up to four failed pharmacologic treatment regimens) and the clinical efficacy demonstrated in prior analyses may not be reflective of the true clinical efficacy of the treatment, as patients with high levels of medication resistance fare worse in their remission/response rates and become more treatment resistant with each succeeding unsuccessful antidepressant trial [13,43,44,45]. Depressed patients with the lowest levels of antidepressant treatment resistance have the greatest likelihood of achieving full remission with rTMS [46].

Most prior evaluations of rTMS in high quality studies (RCTs and systematic review and meta-analyses) have examined its efficacy after multiple (≥2) pharmacologic therapies [7,29]; although some high quality studies (other than the NIMH study noted above) have demonstrated rTMS efficacy after 1–2 failed regimens of pharmacologic medications [35,47,48]. The relative benefits of rTMS vs. pharmacologic medications (when both were compared to placebo) have also demonstrated clinical efficacy with rTMS in a relatively treatment-naïve population [49]. The models of the cost savings and improved health outcomes provided by rTMS provide further evidence supporting the use of rTMS earlier in the treatment process for those patients willing to commit the time to the treatment and those either not willing to take antidepressant medication or; have contraindications to these drugs.

## Limitations

The conclusions drawn from these Markov models should be interpreted in the context of certain limitations. First, estimates of costs, probabilities, and QALYs were extrapolated over the course of the lifetime of a patient based upon shorter term data. While these extrapolations are similar to those in other published studies [8], they may lead to either an underestimate or overestimate of the cost-effectiveness of rTMS.

Second, the costs used in the model were Medicare national average reimbursement rates for procedures/services. While for hospital visits, Medicare on average pays approximately 93% of the costs incurred [50], the relationship to the actual costs of delivering rTMS treatment in the community is not firmly established and may underestimate actual direct costs. Third, adverse event costs, probabilities, and dis-utilities were not included in this analysis. The assumption was that they were similar for each therapy. Nguyen et al [8] estimated dis-utilities for rTMS and ECT with no empirical evidence referenced. Adverse events noted for rTMS in randomized controlled trials included cutaneous scalp discomfort, headache, and pain which diminished rapidly after the first treatment [47,51]. As well, other RCTs found a low discontinuation rate of 4.5% due to mild adverse events [52]. These adverse events did not affect the clinical outcomes [47]. Although the cost and dis-utility effects of adverse events in rTMS likely are minimal, they should be taken into account in future studies. Fourth, these models assume that other direct and indirect costs of MDD were the same for both medication and rTMS treatment. These include the direct costs of concomitant treatment for alcohol and drug abuse [53,54,55], sleep and mood disorders, anxiety, and Attention-Deficit Hyperactivity Disorder (ADHD), and sexual disorders, as well as the indirect costs of disability [56] and suicide [57]. These models also assume other direct non-depression costs are the same which include: non-depression related prescription drugs (@ $1,440 /year) [31] and; non-mental health provider visits (@$7,700/year) [31].

These models assume that patients using antidepressant medications were adherent to their treatment regimens. A lack of adherence would affect costs and possibly decrease quality of life outcomes of treatment.

Lastly, based on the data used, the results would apply to the United States MDD mainly white population. Thus the findings are limited to this group.

## Conclusion

Markov modeling comparing rTMS and medication treatment outcomes indicates that given current practice standards and costs of MDD treatment, rTMS can be a dominant therapy (delivered at lower costs and a higher NMB with better quality of life outcomes) as compared with antidepressant medication treatment. These models indicate that introduction of rTMS treatment after a single failed antidepressant trials would incur greater cost savings and better outcomes than the current practice of continued successive medication trials. Even under less favorable assumptions, rTMS would be a cost-effective alternative based on ICER threshold guidelines. Thus rTMS should be considered by payers for coverage as an MDD treatment earlier in the course of treatment of adults with MDD.

## Supporting information

S1 Fig Cost-effectiveness analysis graph mid 20’s. (PDF)

S2 Fig Incremental cost scatterplot mid 20’s. (PDF)

S3 Fig Incremental cost scatterplot mid 30’s. (PDF)

S4 Fig Incremental cost scatterplot mid 40’s. (PDF)

S5 Fig Incremental cost scatterplot mid 50’s. (PDF)

S6 Fig Tornado diagram–sensitivity analysis mid 20’s. (PDF)

S7 Fig Tornado diagram–sensitivity analysis mid 30’s. (PDF)

S8 Fig Tornado diagram–sensitivity analysis mid 40’s. (PDF)

S9 Fig Tornado diagram–sensitivity analysis mid 50’s. (PDF)

S10 Fig One way sensitivity analysis per session costs rTMS mid 20’s. (PDF)

S11 Fig One way sensitivity analysis monthly cost antidepressant medications mid 20’s. (PDF)

S12 Fig One way sensitivity analysis number of rTMS sessions per year mid 20’s. (PDF)

S13 Fig One way sensitivity analysis per session costs rTMS mid 30’s. (PDF)

S14 Fig One way sensitivity analysis monthly cost antidepressant medications mid 30’s. (PDF)

S15 Fig One way sensitivity analysis number of rTMS sessions per year mid 30’s. (PDF)

S16 Fig One way sensitivity analysis per session costs rTMS mid 40’s. (PDF)

S17 Fig One way sensitivity analysis monthly cost antidepressant medications mid 40’s. (PDF)

S18 Fig One way sensitivity analysis number of rTMS sessions per year mid 40’s. (PDF)

S19 Fig One way sensitivity analysis per session costs rTMS mid 50’s. (PDF)

S20 Fig One way sensitivity analysis monthly cost antidepressant medications mid 50’s. (PDF)

S21 Fig One way sensitivity analysis number of rTMS sessions per year mid 50’s. (PDF)

S1 Appendix Variables used in Markov model. (DOCX)

S2 Appendix Distributions used in Markov model. (DOCX)

S3 Appendix Definitions of terms used in the model. (DOCX)

S4 Appendix rTMS outcomes. (DOCX)

S5 Appendix Drug therapy outcomes. (DOCX)

S6 Appendix Equations used in deriving values in the Markov model. (DOCX)
