# Supplementary figures and images for: Cost effectiveness analysis comparing repetitive transcranial magnetic stimulation to antidepressant medications after a first treatment failure for major depressive disorder in newly diagnosed patients – A lifetime analysis
Source: PLoS One. 2017 Oct 26;12(10):e0186950. doi: 10.1371/journal.pone.0186950 (PMC5658110; doi:10.1371/journal.pone.0186950)

S1 Fig

# Cost-Effectiveness Analysis mid 20s

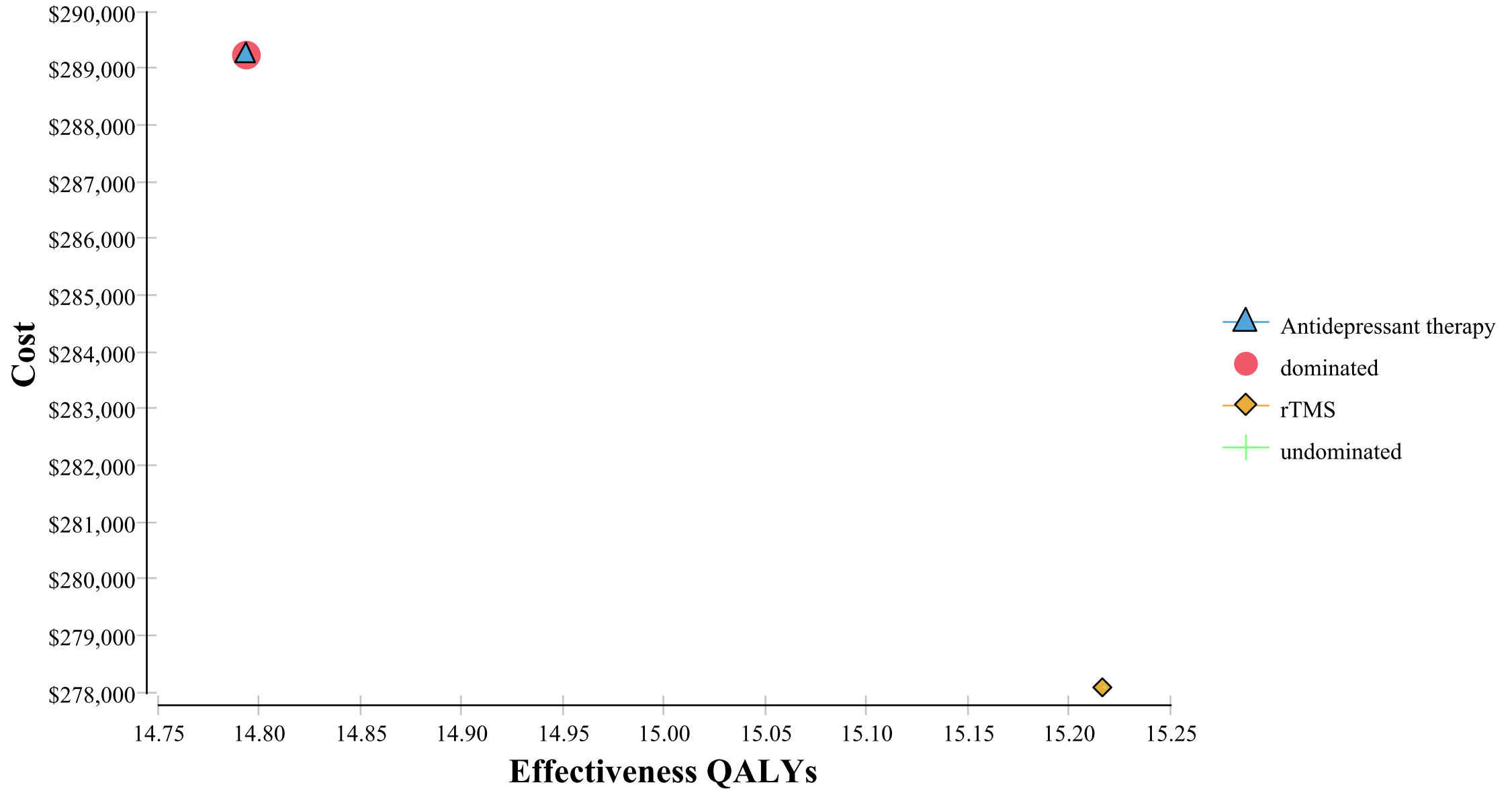

Supplement: S1 Fig — (PDF) [file pone.0186950.s001.pdf]

# Incremental Cost-Effectiveness, rTMS v. Antidepressant therapy mid 20s

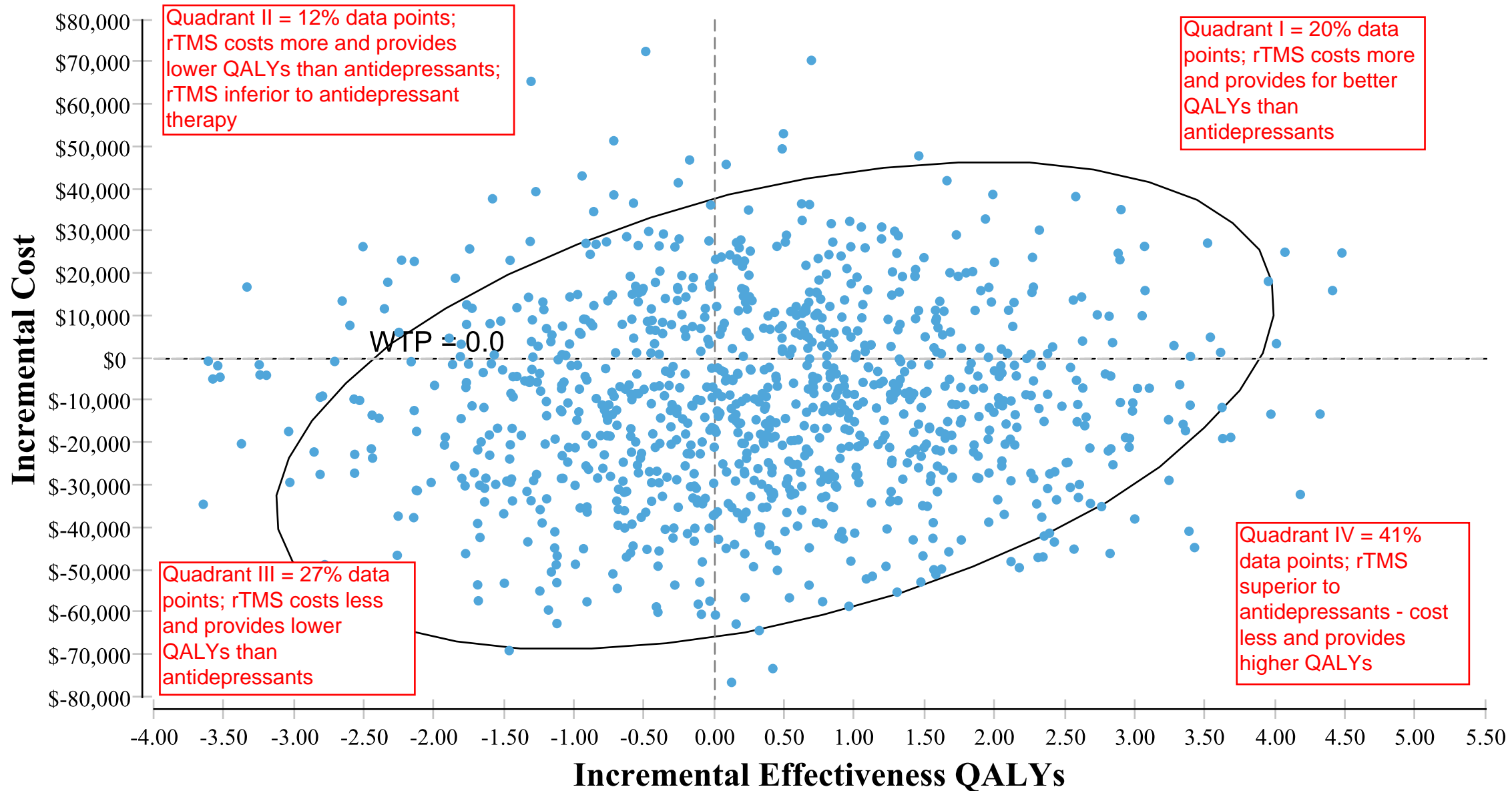

Supplement: S2 Fig — (PDF) [file pone.0186950.s002.pdf]

S3 Fig

Incremental Cost-Effectiveness, rTMS v. Antidepressant therapy mid 30s

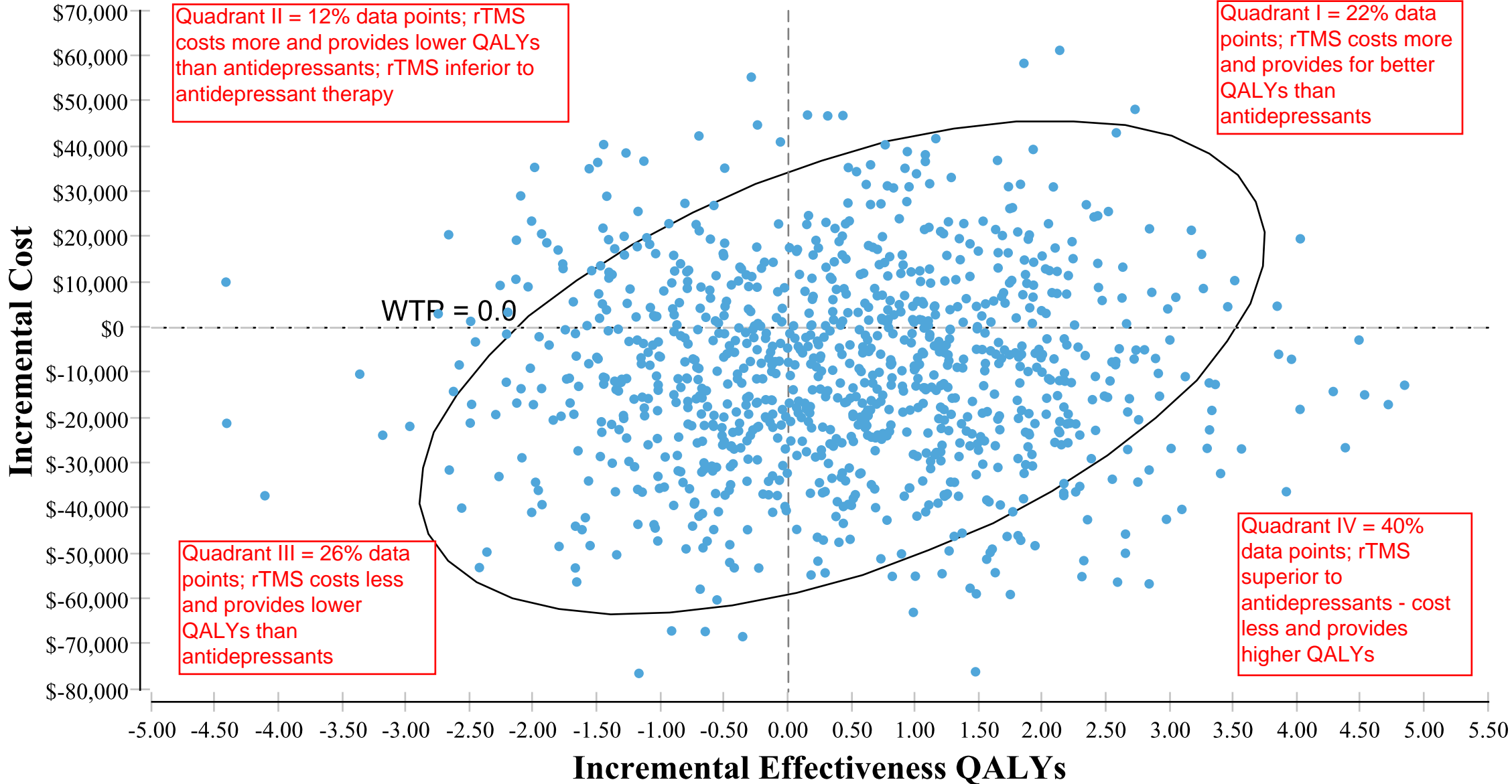

Supplement: S3 Fig — (PDF) [file pone.0186950.s003.pdf]

## Incremental Cost-Effectiveness, rTMS v. Antidepressant therapy mid 40s

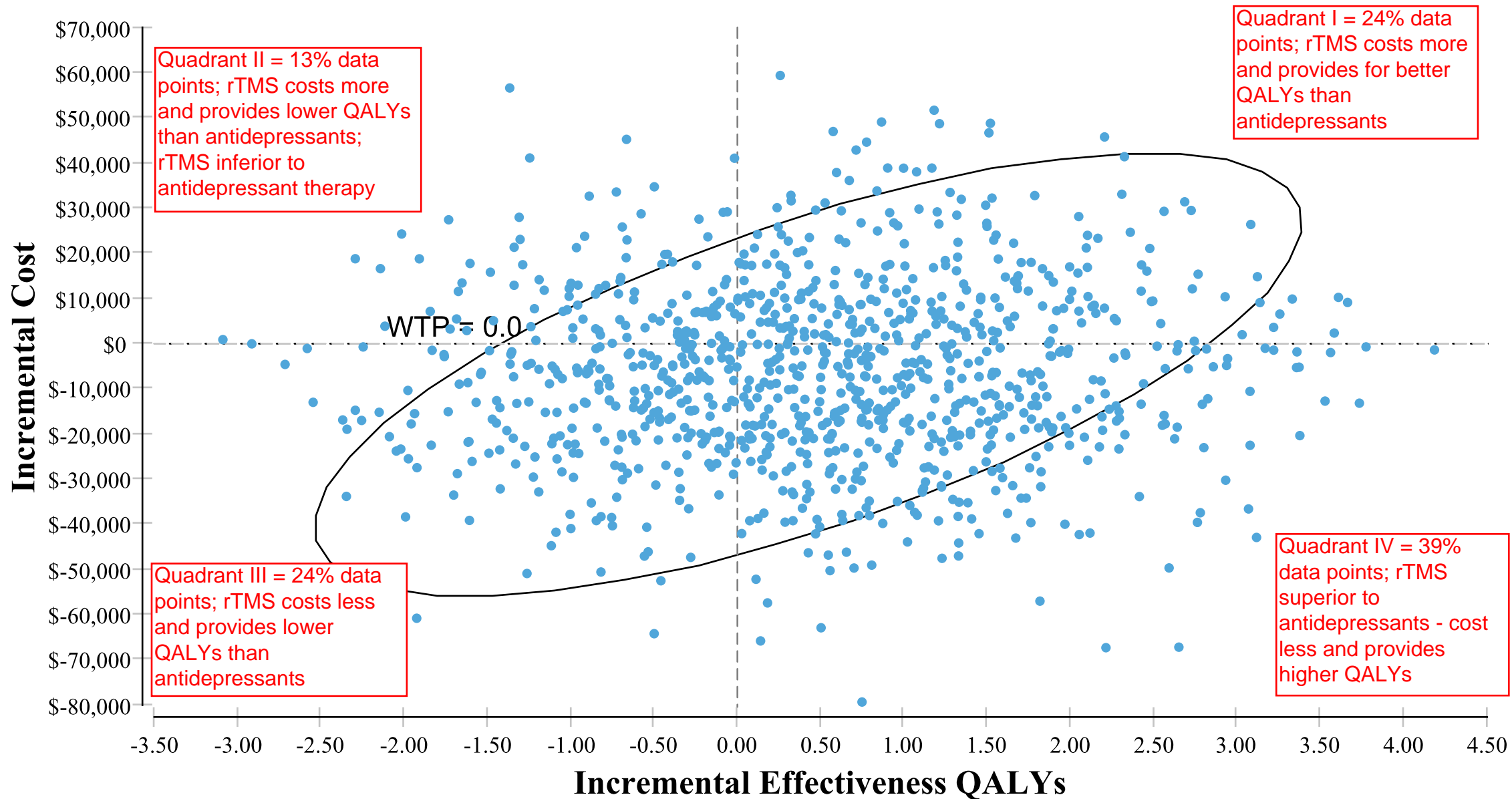

Supplement: S4 Fig — (PDF) [file pone.0186950.s004.pdf]

# Incremental Cost-Effectiveness, rTMS v. Antidepressant therapy mid 50s

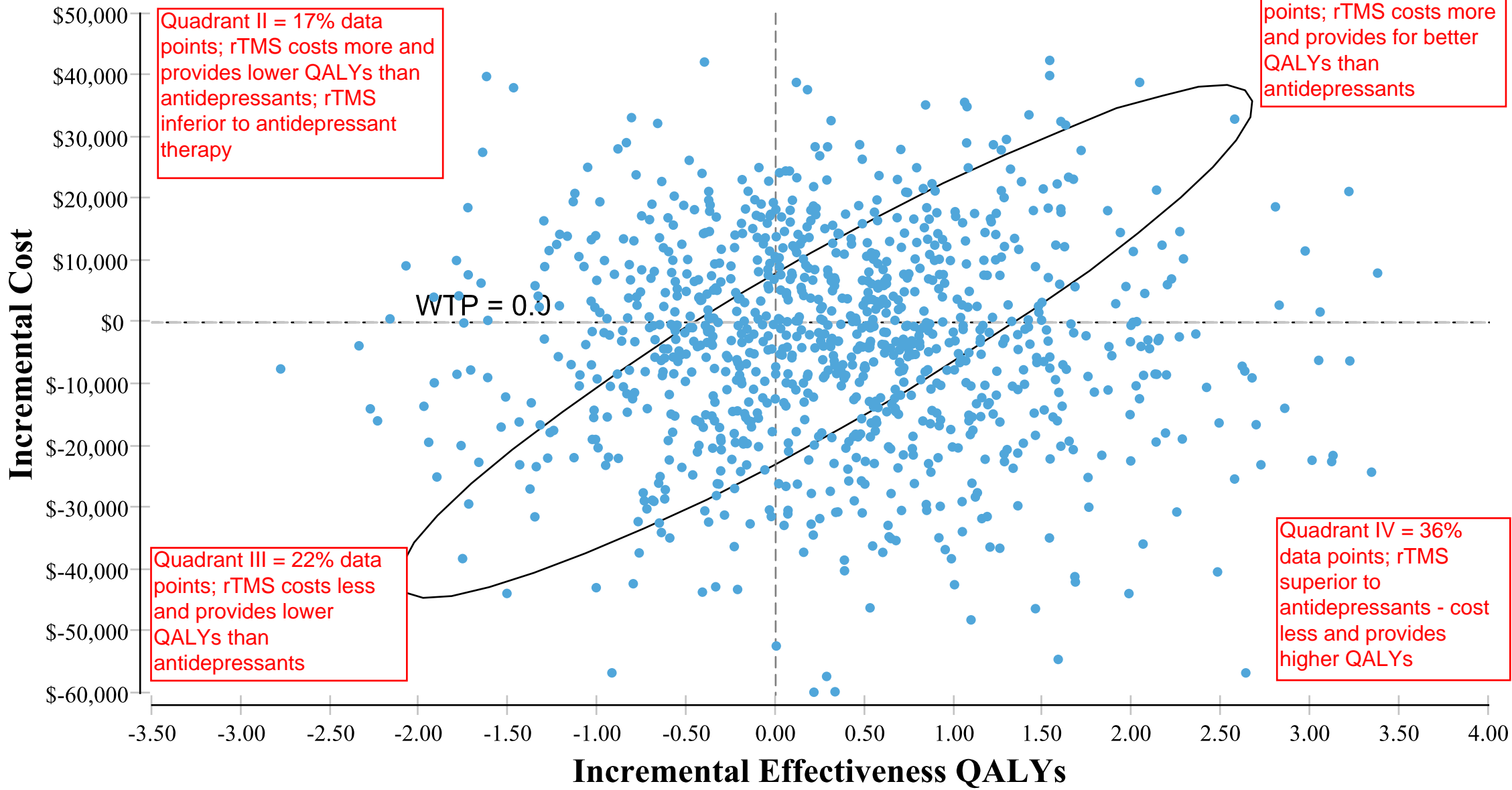

Supplement: S5 Fig — (PDF) [file pone.0186950.s005.pdf]

## Tornado Analysis (Net Benefits) mids 30s

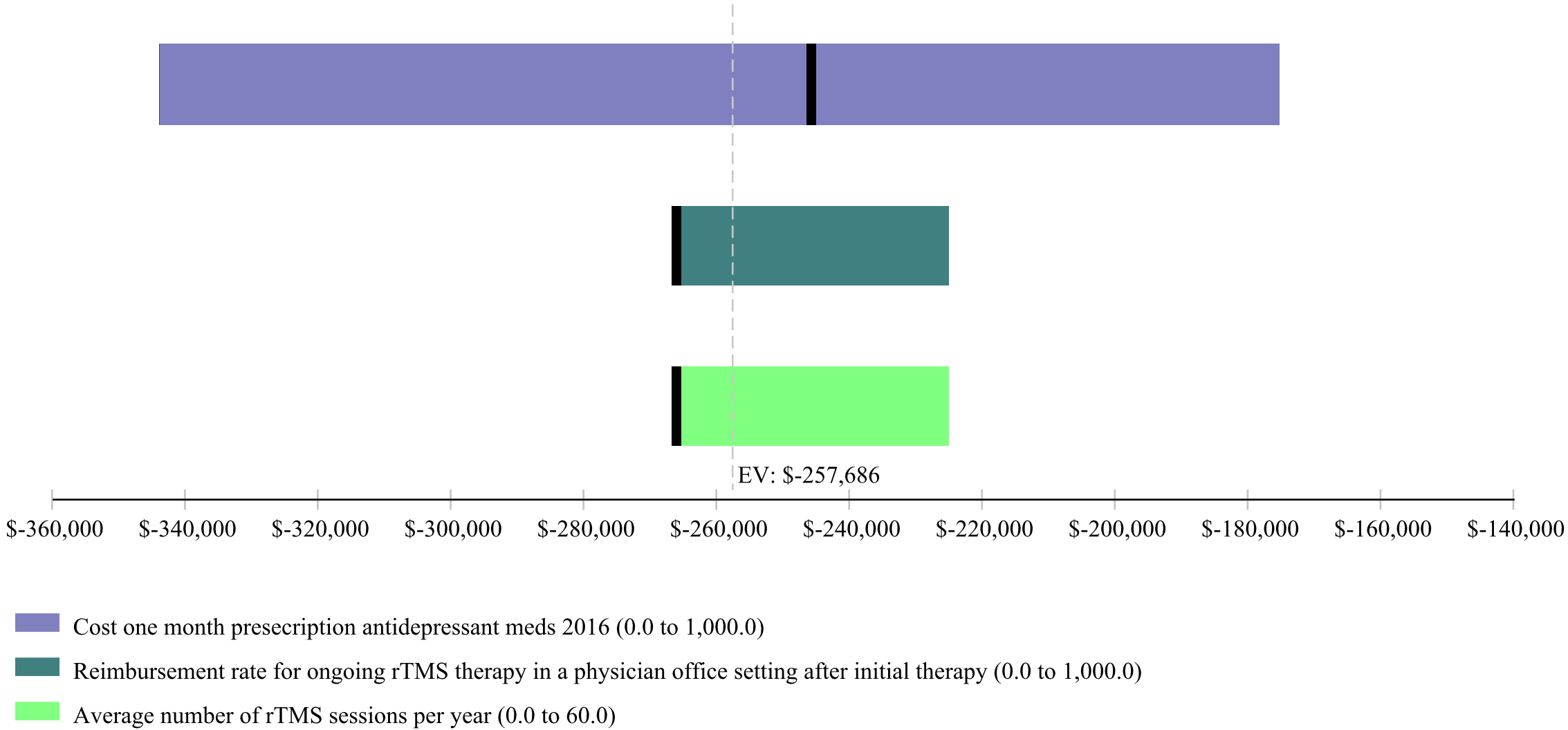

Supplement: S7 Fig — (PDF) [file pone.0186950.s007.pdf]

Tornado Analysis (Net Benefits) - age mid 50s

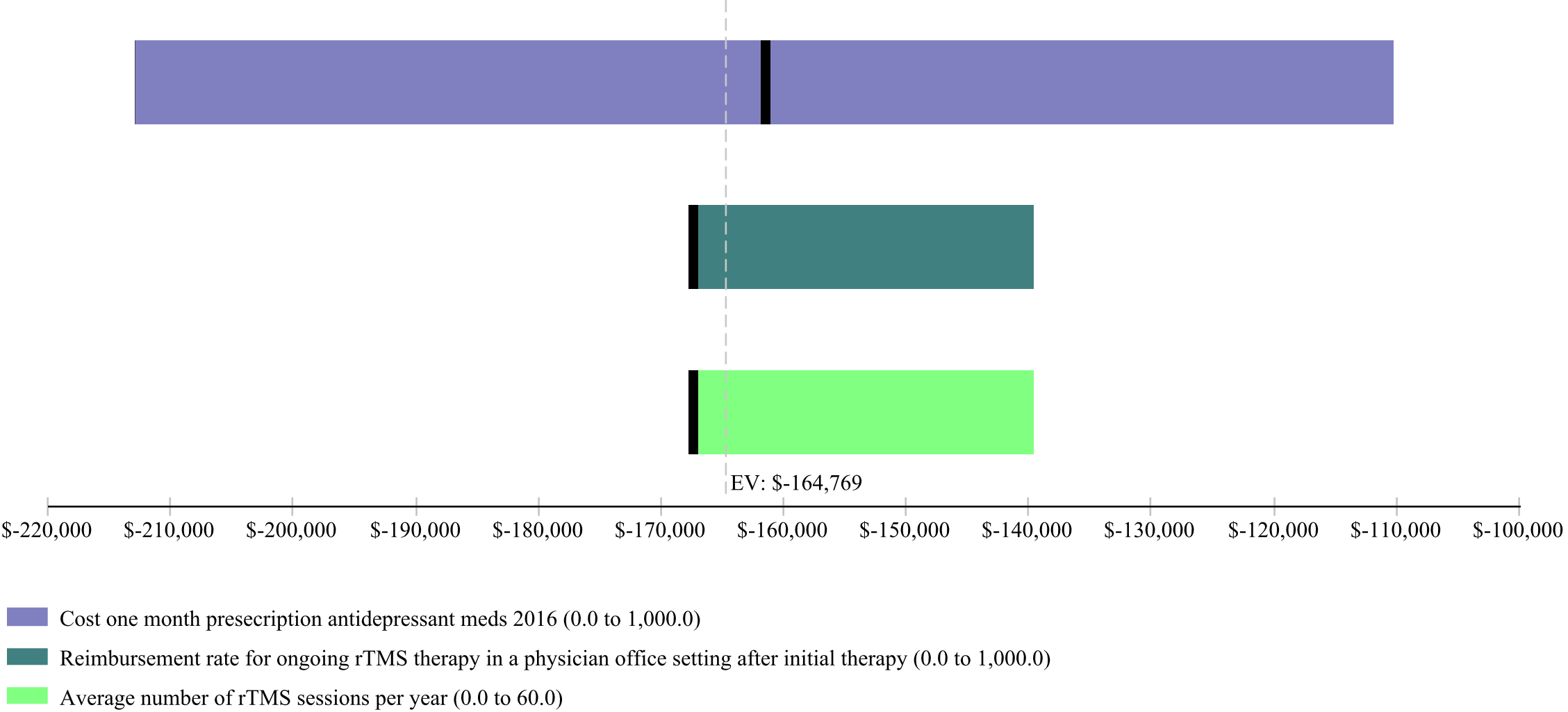

Supplement: S9 Fig — (PDF) [file pone.0186950.s009.pdf]

Sensitivity Analysis per session cost rTMS mid 20s

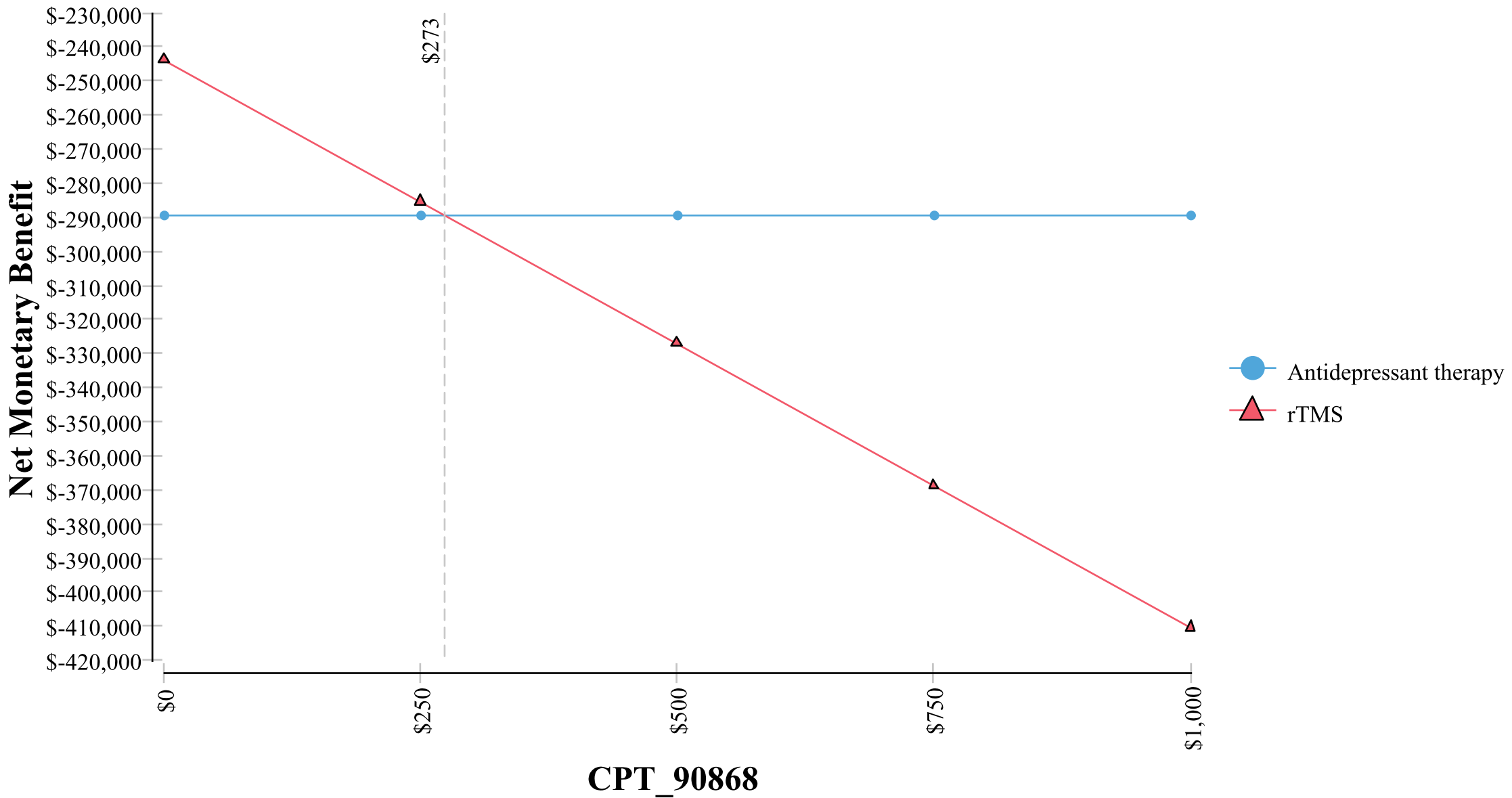

Supplement: S10 Fig — (PDF) [file pone.0186950.s010.pdf]

**S11 Fig**

**Sensitivity Analysis monthly cost antidepressant meds mid 20s**

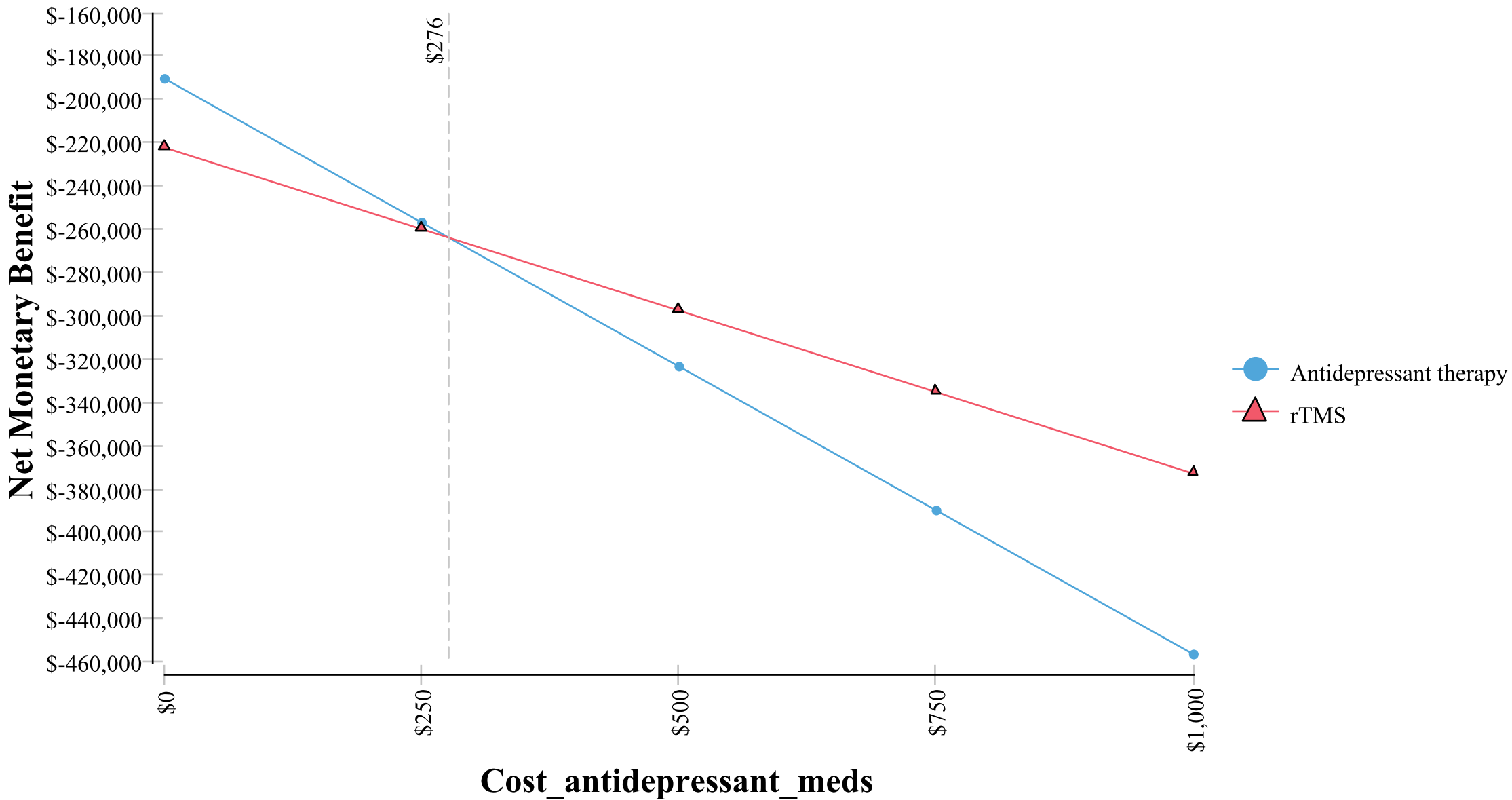

Supplement: S11 Fig — (PDF) [file pone.0186950.s011.pdf]

Sensitivity Analysis number rTMS sessions mid 20s

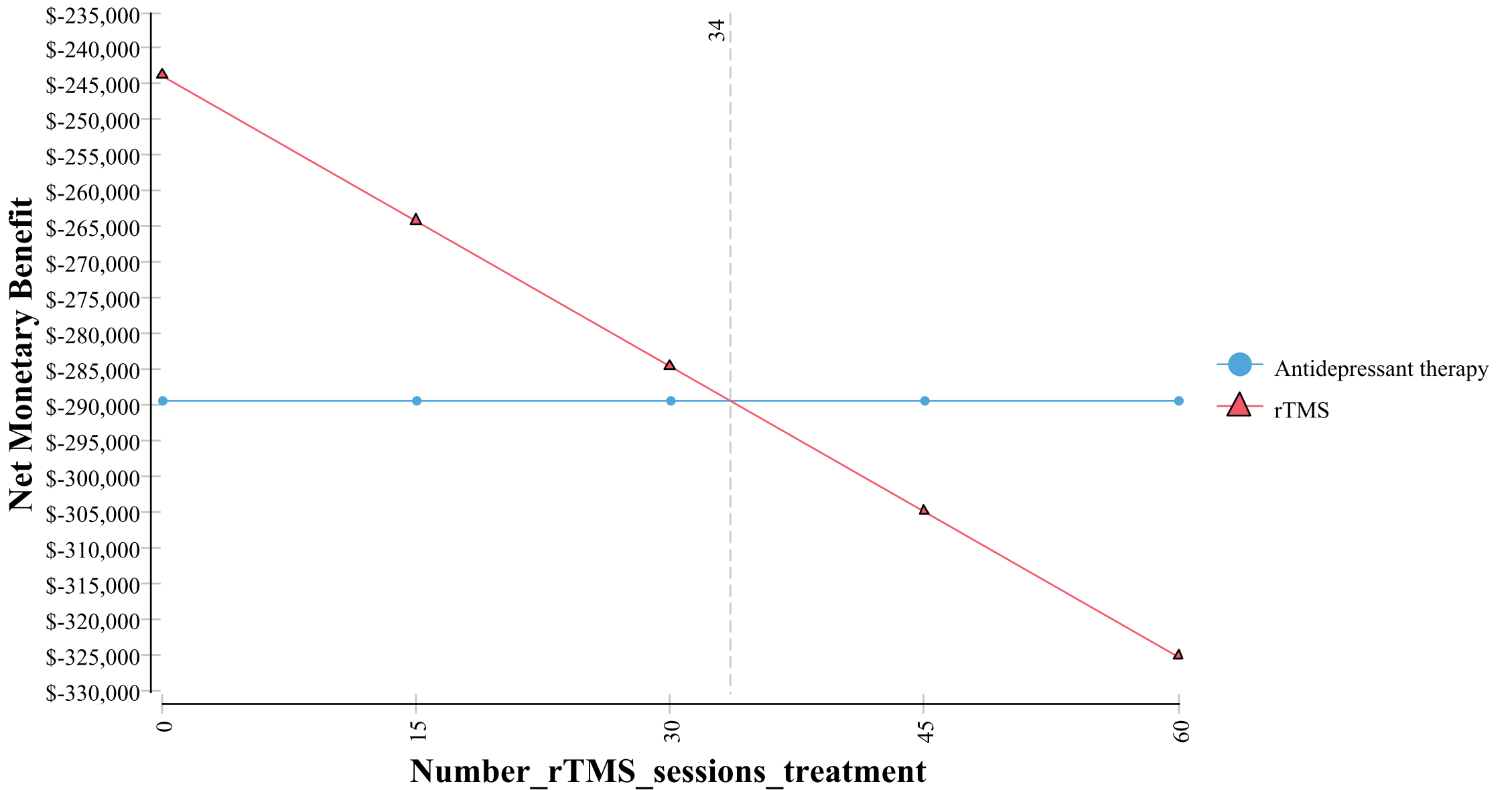

Supplement: S12 Fig — (PDF) [file pone.0186950.s012.pdf]

Sensitivity Analysis per session cost rTMS mid 30s

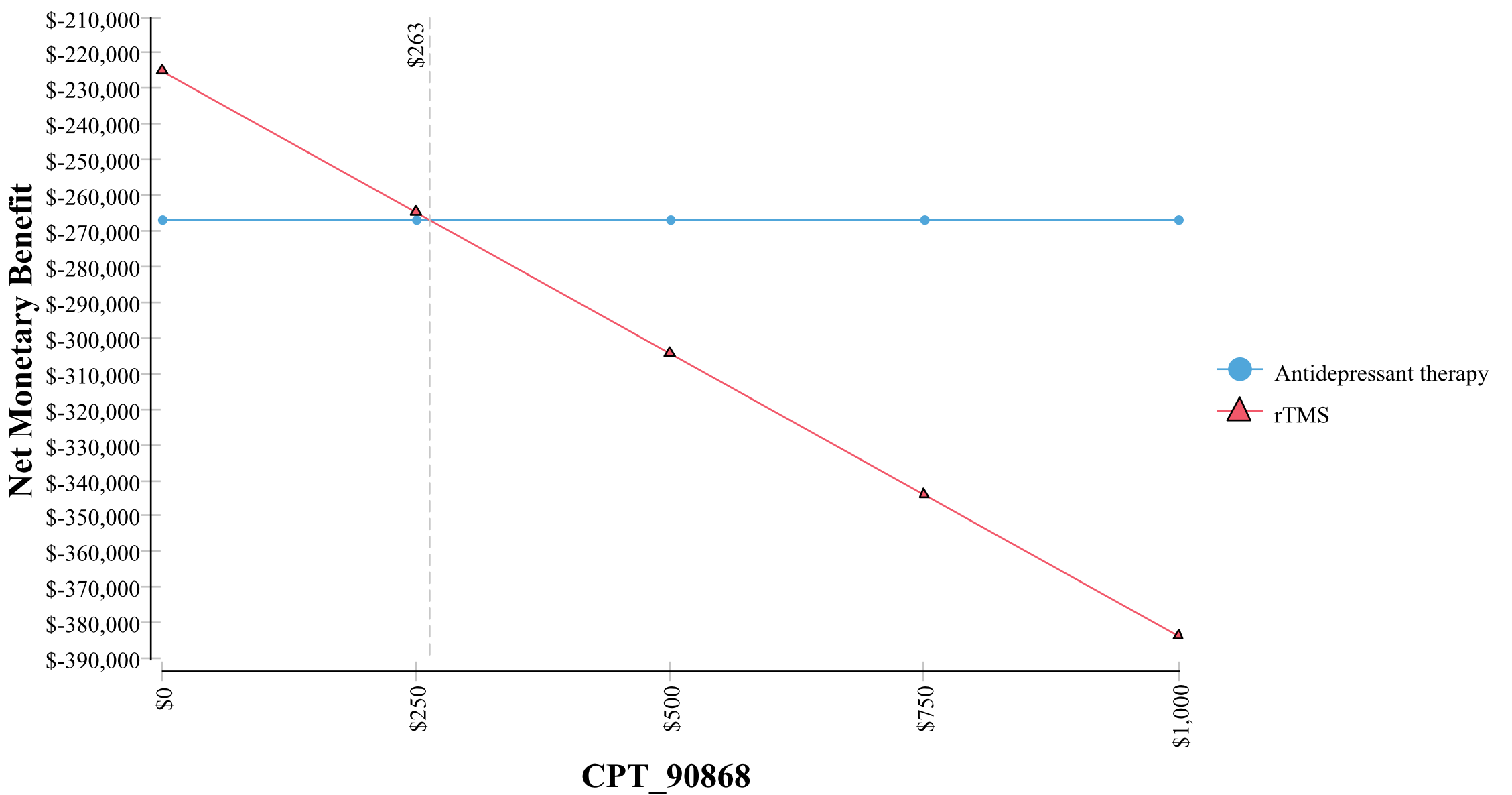

Supplement: S13 Fig — (PDF) [file pone.0186950.s013.pdf]

**S14 Fig**

**Sensitivity Analysis monthly cost antidepressant meds mid 30s**

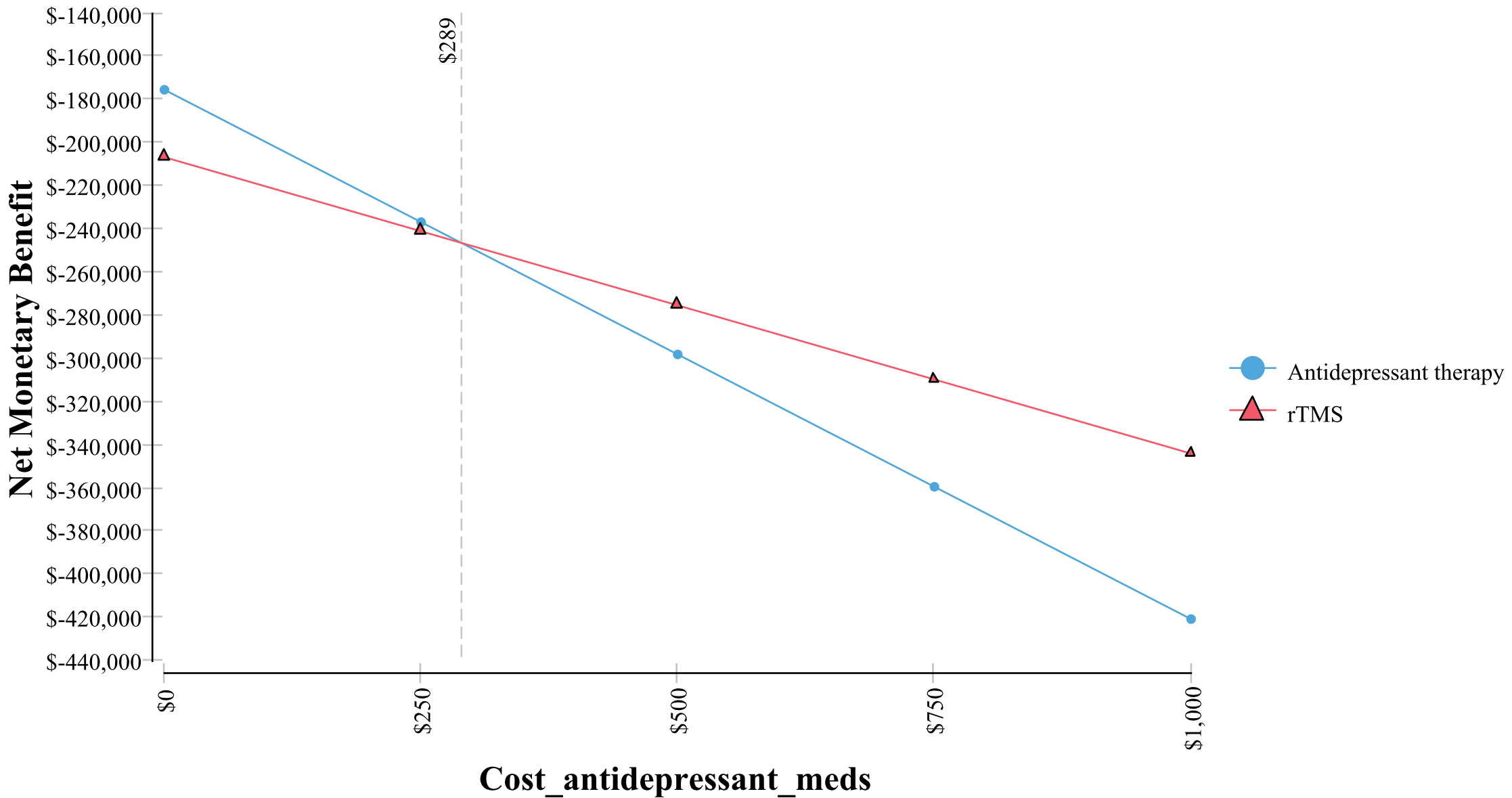

Supplement: S14 Fig — (PDF) [file pone.0186950.s014.pdf]

# Sensitivity Analysis number rTMS sessions mid 30s

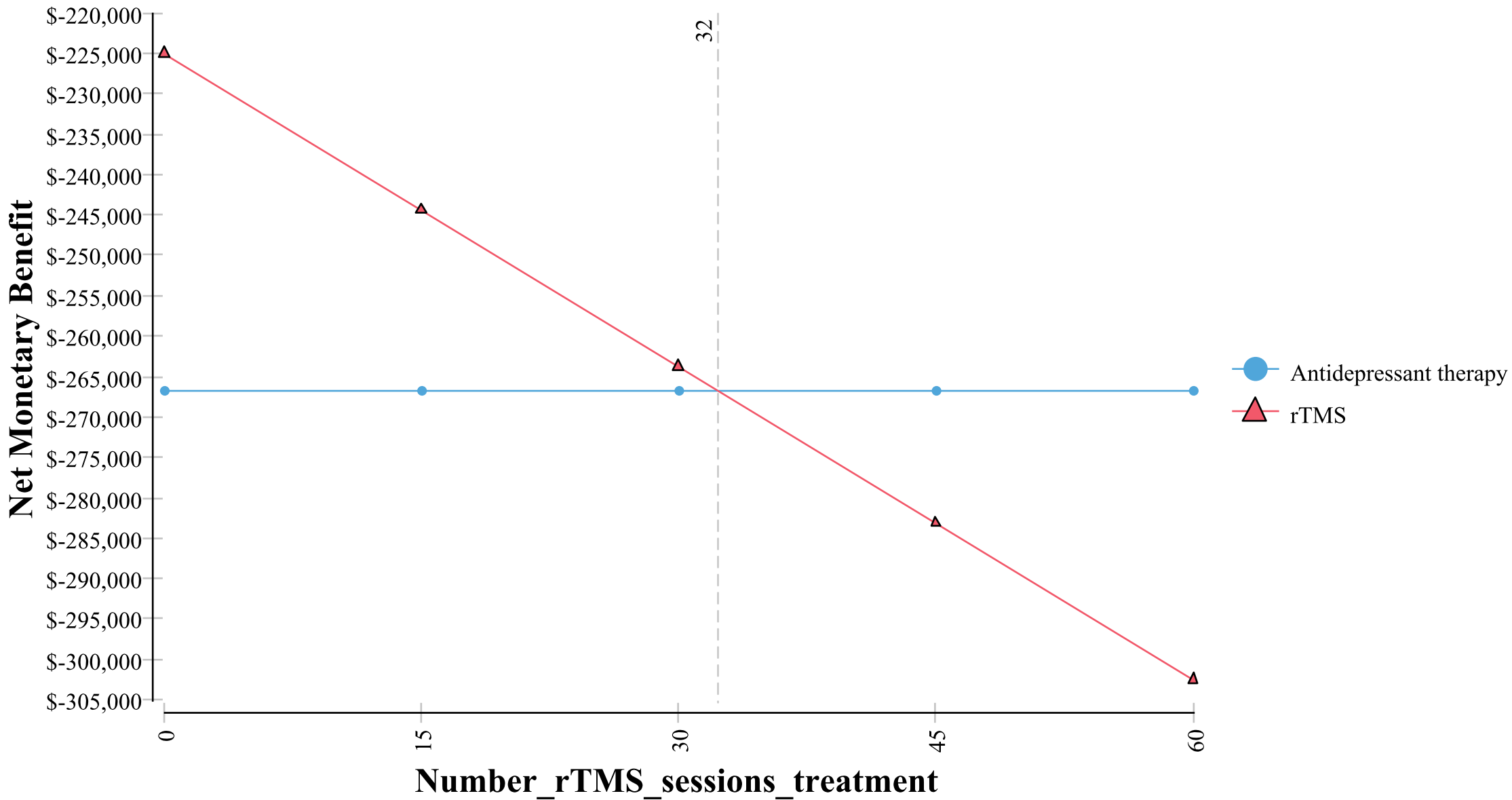

Supplement: S15 Fig — (PDF) [file pone.0186950.s015.pdf]

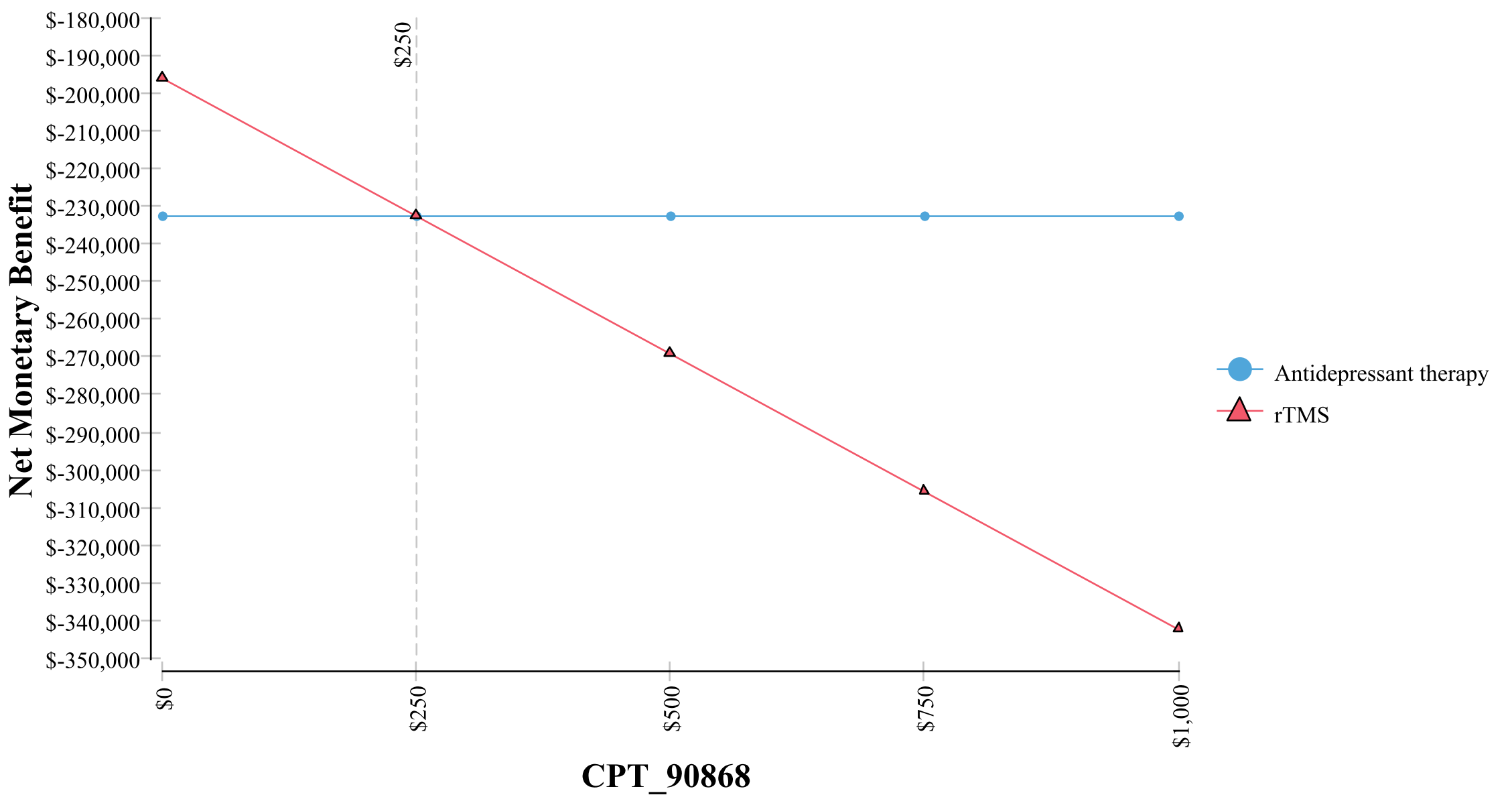

Supplement: S16 Fig — (PDF) [file pone.0186950.s016.pdf]

Sensitivity Analysis monthly cost antidepressant meds mid 40s

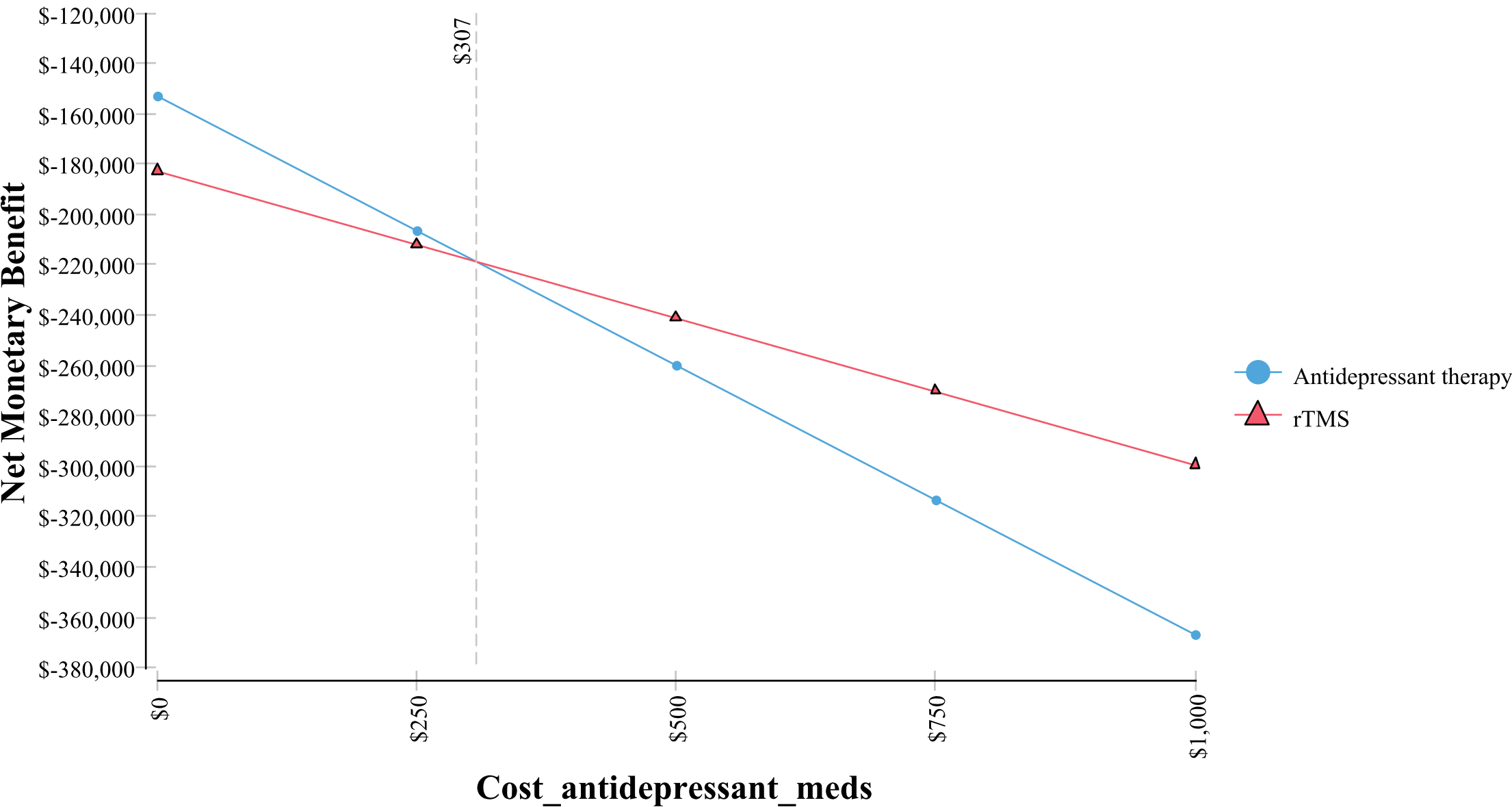

Supplement: S17 Fig — (PDF) [file pone.0186950.s017.pdf]

Sensitivity Analysis number rTMS sessions mid 40s

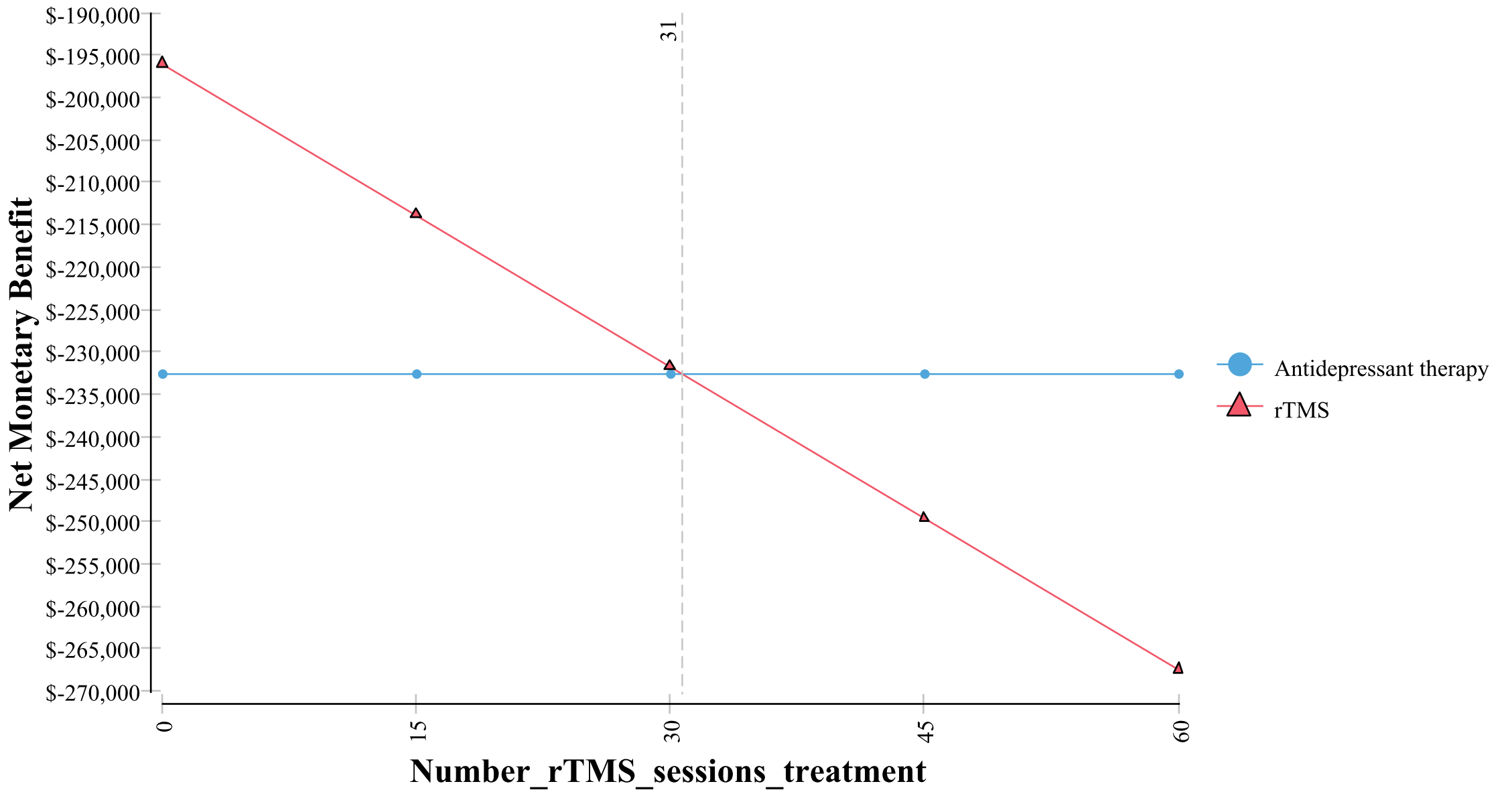

Supplement: S18 Fig — (PDF) [file pone.0186950.s018.pdf]

Sensitivity Analysis per session cost rTMS mid 50s

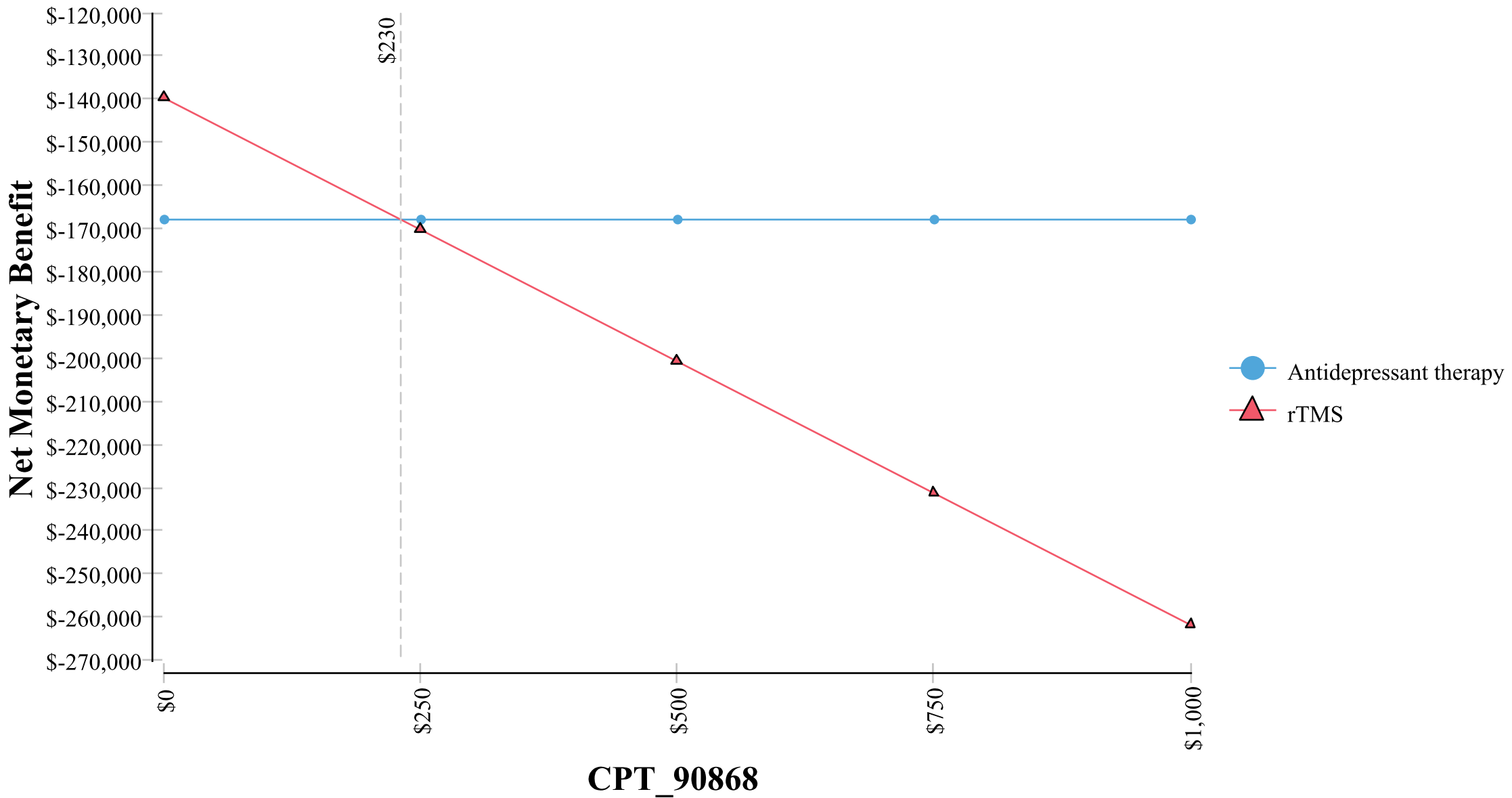

Supplement: S19 Fig — (PDF) [file pone.0186950.s019.pdf]

Sensitivity Analysis - monthly cost antidepressant meds - mid 50s

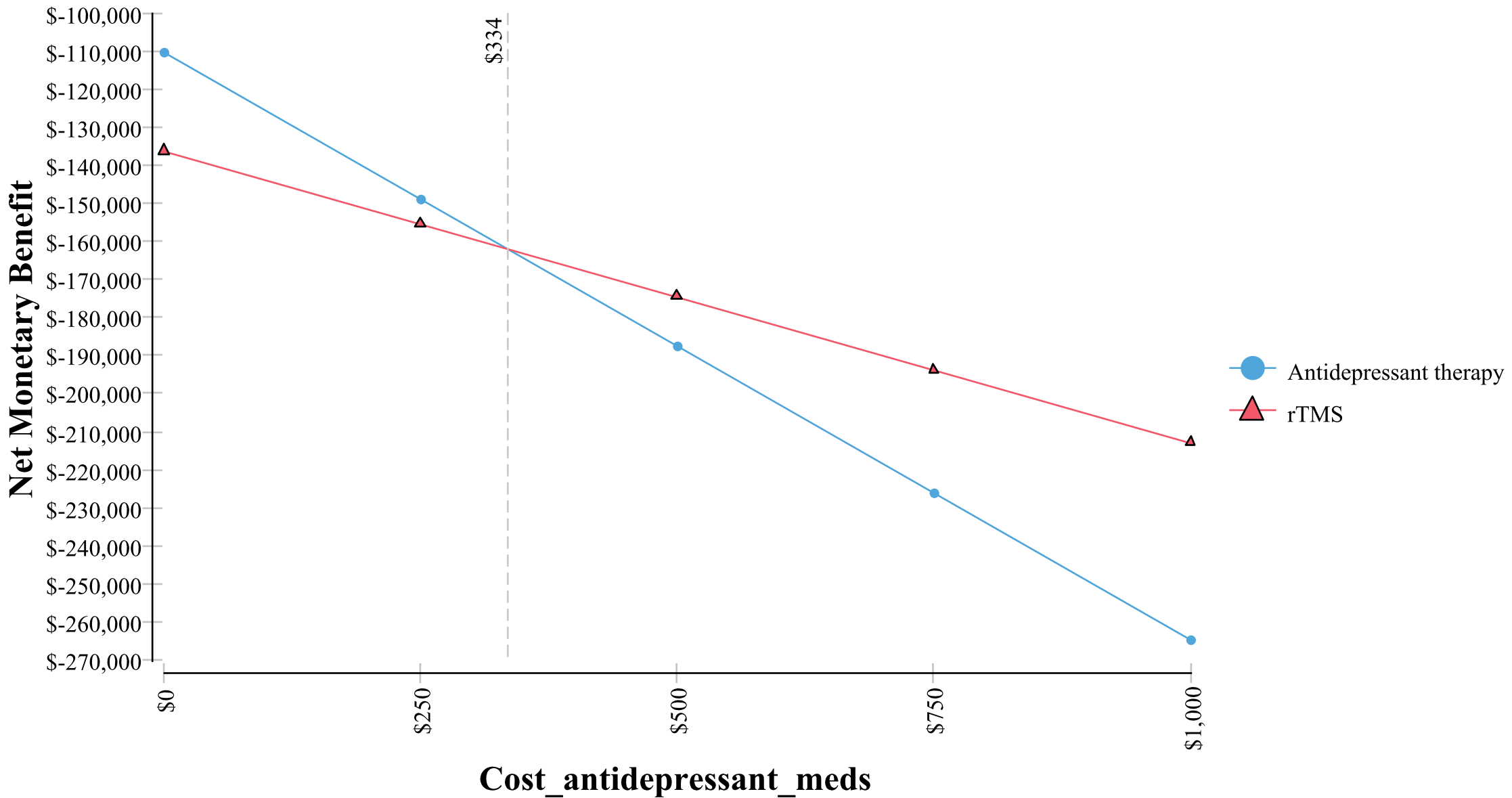

Supplement: S20 Fig — (PDF) [file pone.0186950.s020.pdf]

Sensitivity Analysis number rTMS sessions mid 50s

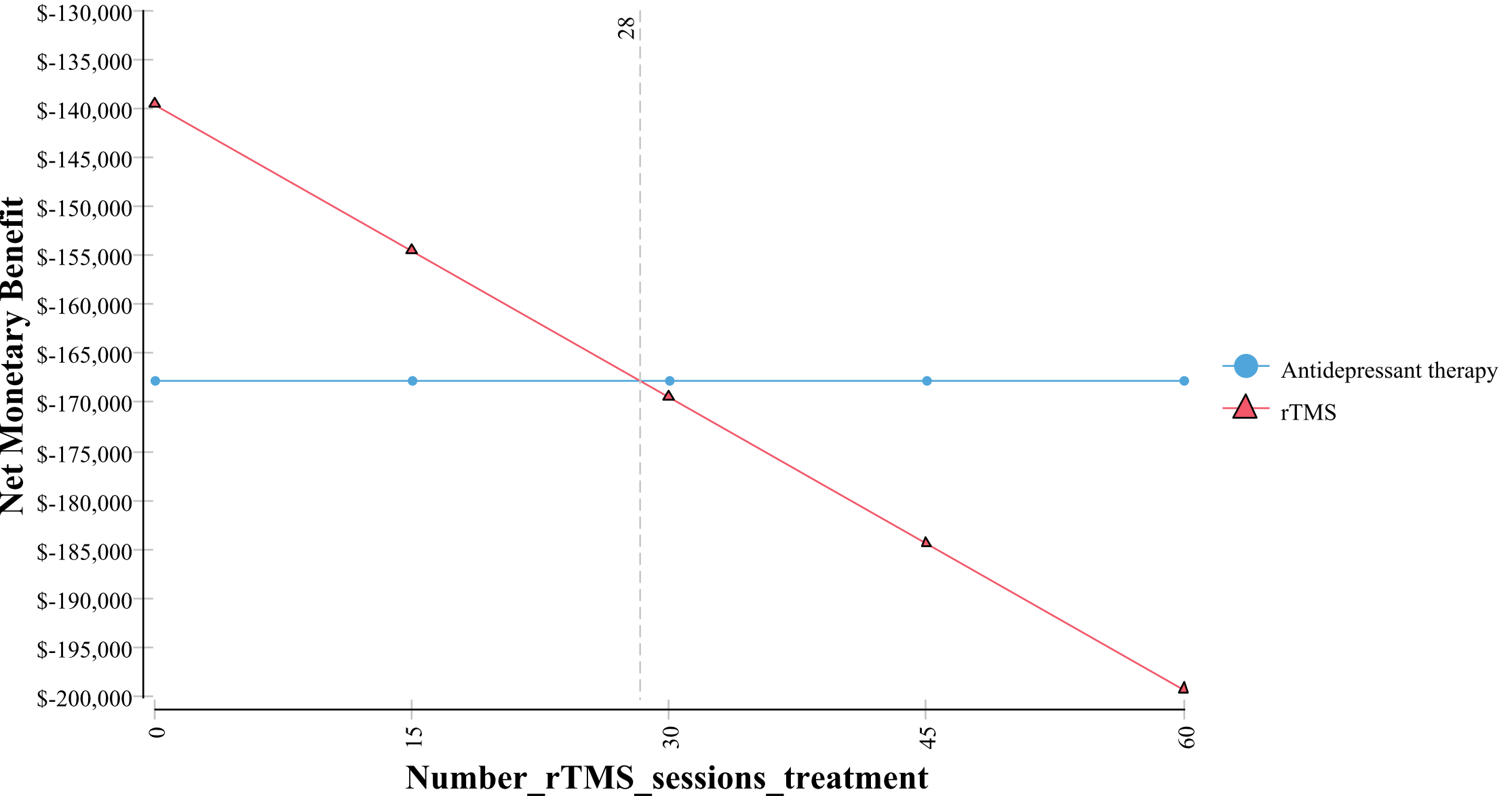

Supplement: S21 Fig — (PDF) [file pone.0186950.s021.pdf]
