# Supplementary material for: Cost effectiveness analysis comparing repetitive transcranial magnetic stimulation to antidepressant medications after a first treatment failure for major depressive disorder in newly diagnosed patients – A lifetime analysis
Source: PLoS One. 2017 Oct 26;12(10):e0186950. doi: 10.1371/journal.pone.0186950 (PMC5658110; doi:10.1371/journal.pone.0186950)
Supplement: S8 Fig — (PDF) [file pone.0186950.s008.pdf]

S8 Fig

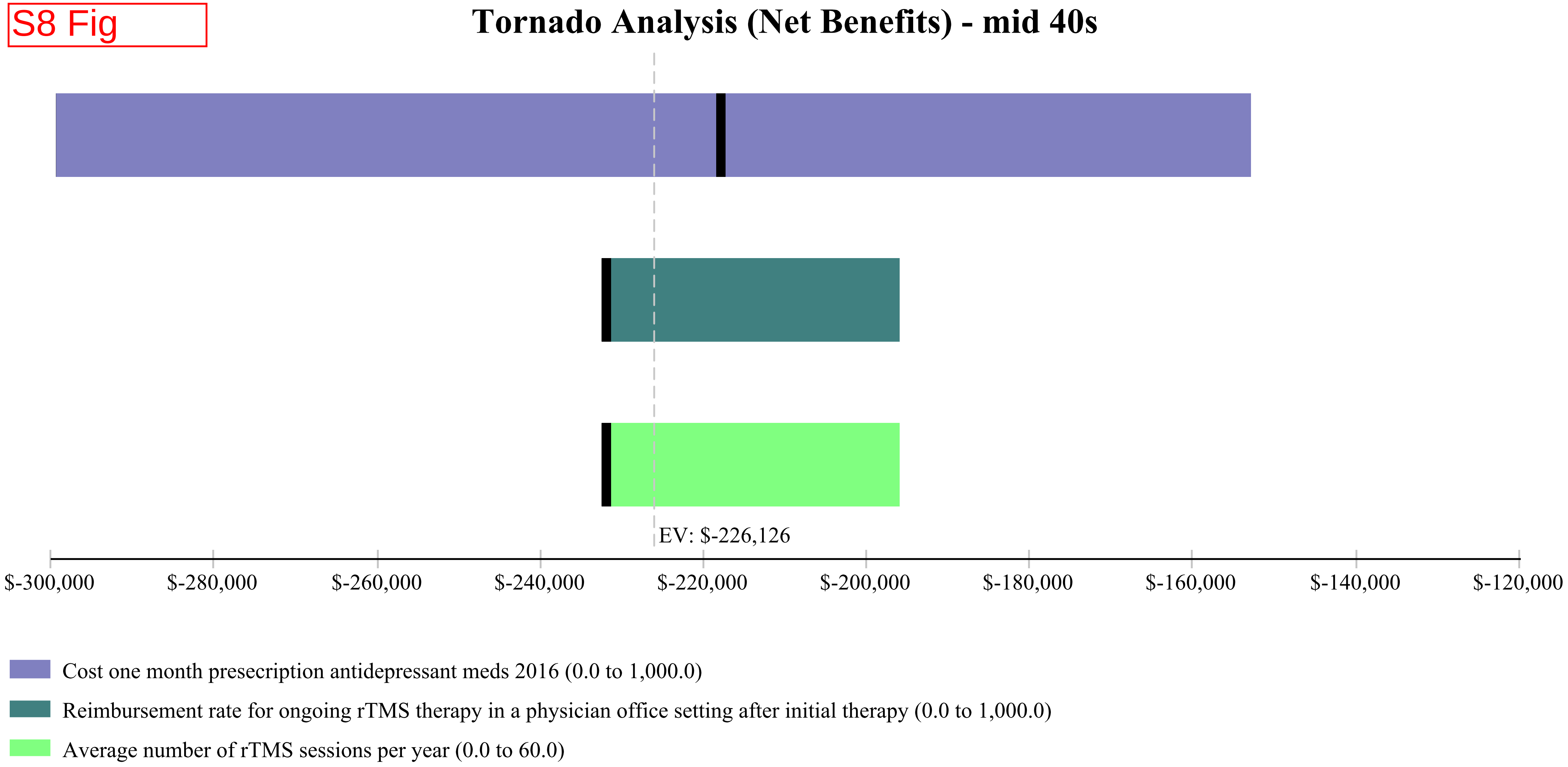
Tornado Analysis (Net Benefits) - mid 40s
EV: $-226,126
$-300,000
$-280,000
$-260,000
$-240,000
$-220,000
$-200,000
$-180,000
$-160,000
$-140,000
$-120,000
Cost one month presecription antidepressant meds 2016 (0.0 to 1,000.0)
Reimbursement rate for ongoing rTMS therapy in a physician office setting after initial therapy (0.0 to 1,000.0)
Average number of rTMS sessions per year (0.0 to 60.0)
