# Supplementary material for: Cost effectiveness analysis comparing repetitive transcranial magnetic stimulation to antidepressant medications after a first treatment failure for major depressive disorder in newly diagnosed patients – A lifetime analysis
Source: PLoS One. 2017 Oct 26;12(10):e0186950. doi: 10.1371/journal.pone.0186950 (PMC5658110; doi:10.1371/journal.pone.0186950)
Supplement: S1 Appendix — (DOCX) [file pone.0186950.s022.docx]

S1 Appendix: Variables used in model

| **NAME** | **DESCRIPTION** | **FORMULA** | **VALUE** | **LOW** | **HIGH** | **COMMENT** |
| --- | --- | --- | --- | --- | --- | --- |
| APC_5723 | Payment for ambulatory payment classification (APC) 5723 - electroconvulsive therapy. | $397 | 397.0 | 0 | 397 | Amount paid to hospital for ECT during an outpatient setting |
| Cost_antidepressant_meds | Cost one month prescription antidepressant meds 2016 | $372.50 | 372.5 | 0 | 500 | Derived from: Builema A, et al. Impact of co-payment discounts on antidepressant medication adherence and costs. Am Jr. Pharm Benefits.  2015;7(3):136-142. 2010 costs per year of $3,760 were adjusted by prescription CPI to the year 2016. @ $4,470/year or $372.50/month |
| Cost_Medical_Services_yearly | Cost of additional medical services per year | $1,465 | 1465.0 | 0 | 1465 | Greenberg PE, et al. The economic burden of adults with major depressive disorder in the United States (2005 and 2010). Journal Clinical Psychiat 2015. 76(2):155-162. Includes: inpatient, ED, outpatient. Dollars inflated from 2012. 2012 $ = $1,289; 2016 $ = $1,465. Note: This amount in 2012 does not include the Other category which amounted to $121/yr. This amount was not included and was assumed to include such services as rTMS and ECT. Therefore it was excluded. |
| CPT_00104 | Anesthesia provided to patient during ECT session | $98 | 98.0 | 0 | 98 | Assumes base units of 4; one 15 minute session with a conversion factor of $24.50. Therefore: 4 X 1 X $24.50 = $98 |
| CPT_90832 | Psychotherapy 1 hour per week @ $128.48 per week - 2017 Medicare national average amt | $128.48 | 128.48 | 0 | 128.48 | Medicare 2016 payment rate - payment in NYC |
| CPT_90867 | Reimbursement rate for rTMS for initial therapy provided in a physician office setting | $367 | 367.0 | 0 | 367 | Medicare 2016 payment rate - payment in NYC |
| CPT_90868 | Reimbursement rate for ongoing rTMS therapy in a physician office setting after initial therapy | $206 | 206.0 | 0 | 600 | Medicare 2016 payment rate - NYC |
| CPT_90869 | Reimbursement rate of subsequent motor threshold redetermination with delivery and management | $200 | 200.0 | 0 | 200 | Medicare 2016 payment rate - NYC |
| CPT_90870 | Reimbursement rate for physician service provided during electroconvulsive therapy | $112 | 112.0 | 0 | 112 | Medicare 2016 payment rate for the non-facility setting |
| CPT_99214 | Reimbursement rate Medicare for an evaluation and management of patient - established | $108.13 | 108.13 | 0 | 108.13 | 2016 payment rate for Medicare for E&M (99214) - 45 minute evaluation of a patient - nonfacility setting |
| Number_ECT_sessions | Number of treatment sessions with ECT initial | Number_treatment_sessions_ECT | 9.0 | 0 | 9 | Derived from 2016 Aetna coverage policy for ECT |
| Number_rTMS_sessions_maintenance | Number of rTMS maintenance sessions per year | Number_rTMS_maintenance_sessions | 4.0 | 0 | 40 | Nguyen et al. 2015 Value in Health - expert opinion |
| Number_rTMS_sessions_treatment | Average number of rTMS sessions per year | Number_treatment_sessions_rTMS | 25.3 | 8.6 | 42 | Philip NS, et al. Can medication free, treatment-resistant, depressed patients who initially respond to TMS be maintained off medications? A prospective, 12-month multisite randomized pilot trial. Brain Stimulation. 2016 9(20):251-7. |
| Probability_death_MDD | Probability death per year from major depressive disorder | Probability_death_MDD_year | 0.02 | 0 | 0.02 | Vythilingam M, et al. Psychotic depression and mortality. Amer Jrl Psych 2003. 160:574-576. |
| Probability_relapse_lose_remission_pharma | Probability of relapsing with pharma after remission | Prob_relapse_meds | 0.185 | 0 | 0.185 | Primary Care Companion. Jrl Clin Psych 2007. 9(3):214-223. Note: These are similar findings to: Perahia DG et al. Duloxetine in the prevention of relapse of major depressive disorder. Brit Jrl Psychiat 2006. 188:346-353 which showed at relapse rate of 17.4% - 21.9%. |
| Probability_relapse_lose_remission_rTMS | Probability of relapsing after rTMS over 6 month period | 0.1 | 0.1 | 0 | 0.8 | Janicak PG et al. Durability of clinical benefit with transcranial magnetic stimulation (TMS) in the treatment of pharmacoresistant major depression: assessment of relapse during a 6-month, multisite, open-label study. Brain Stimulation 2010;3:187-199. |
| Probability_remission_ECT_after_second_failed_therapy |  | Prob_remission_other_Rx_with_ECT_failed_second_therapy | 0.45 | 0 | 0.4543 | Nguyen K-H, et al. Cost effectiveness of repetitive transcranial magnetic stimulation versus antidepressant therapy for treatment- resistant depression. Value in Health. 2015. 18:597-604. |
| Probability_remission_first_to_second_meds | Probability of remission after first failed therapy - using second line for medications | 0.92 | 0.92 | 0 | 0.19 | Sinyor M et al. The Sequenced treatment alternatives to relieve depression (STAR*D) trial: A review. Canadian Jrl Psychiatry. 2010;55(3):126-35. See figure 3; Page 132. Note with second line medication therapy in total there were 0.63 responders and 0.58 remitters. Therefore, 0.58/0.63 = 0.92 of responders were remitters |
| Probability_remission_first_to_second_rTMS |  | Remission_rate_first_failure_second_therapy_rTMS | 0.655 | 0 | 0.8 | Nguyen K-H, et al. Cost effectiveness of repetitive transcranial magnetic stimulation versus antidepressant therapy for treatment- resistant depression. Value in Health. 2015. 18:597-604. |
| Probability_remission_second_to_third_meds | Probability of remission after second failed therapy with third line meds | 0.06 | 0.06 | 0 | 0.06 | Sinyor M et al. The Sequenced treatment alternatives to relieve depression (STAR*D) trial: A review. Canadian Jrl Psychiatry. 2010;55(3):126-35. See figure 3; Page 132 |
| Probability_remission_second_to_third_rTMS |  | Remission_rate_second_failure_third_therapy_rTMS | 0.2545 | 0 | 0.8 | Nguyen K-H, et al. Cost effectiveness of repetitive transcranial magnetic stimulation versus antidepressant therapy for treatment- resistant depression. Value in Health. 2015. 18:597-604. |
| Probability_remission_third_to_fourth_rTMS |  | Remission_rate_third_failure_fourth_therapy_rTMS | 0.20 | 0 | 0.2038 | Nguyen K-H, et al. Cost effectiveness of repetitive transcranial magnetic stimulation versus antidepressant therapy for treatment- resistant depression. Value in Health. 2015. 18:597-604. |
| Probability_response_ECT_with_retreatment | Probability of a response with ECT during retreatment | Probability_response_ECT | 0.3722 | 0 | 0.3722 | Vallejo-Torres L, et al. Cost-effectiveness of electroconvulsive therapy compared to repetitive transcranial magnetic stimulation for treatment-resistant severe depression: a decision model. Psych Med 2015. 45:1459-1470. |
| Probability_response_first_to_second_meds | Probability of a response after first failed therapy - with second line meds | 0.13 | 0.13 | 0 | 0.13 | Sinyor M et al. The Sequenced treatment alternatives to relieve depression (STAR*D) trial: A review. Canadian Jrl Psychiatry. 2010;55(3):126-35. See figure 3; Page 132 |
| Probability_response_first_to_second_rTMS |  | Response_rate_first_failure_second_therapy_rTMS | 0.4818 | 0 | 0.9 | Nguyen K-H, et al. Cost effectiveness of repetitive transcranial magnetic stimulation versus antidepressant therapy for treatment- resistant depression. Value in Health. 2015. 18:597-604. |
| Probability_response_second_to_third_meds | Probability of a response after second failed therapy with a third line med | 0.06 | 0.06 | 0 | 0.06 | Sinyor M et al. The Sequenced treatment alternatives to relieve depression (STAR*D) trial: A review. Canadian Jrl Psychiatry. 2010;55(3):126-35. See figure 3; Page 132 |
| Probability_response_second_to_third_rTMS |  | Response_rate_second_to_third_rTMS | 0.4095 | 0 | 0.4095 |  |
| Probability_response_third_to_fourth_meds | Probability of a response after third failed attempt with fourth line meds | 0.04 | 0.04 | 0 | 0.04 | Sinyor M et al. The Sequenced treatment alternatives to relieve depression (STAR*D) trial: A review. Canadian Jrl Psychiatry. 2010;55(3):126-35. See figure 3; Page 132 |
| Probability_response_third_to_fourth_rTMS |  | Response_rate_rTMS_third_to_fourth_rTMS | 0.348 | 0 | 0.348 |  |
| Probability_retreatment_existing_therapy | Probability of physician staying with existing therapy during retreatment | 0.5 | 0.5 | 0 | 0.5 |  |
| QoL_MDD_baseline_drug_therapy | QoL baseline in patients with MDD treated with antidepressants | QoL_baseline_MDD_antidepressants | 0.47 | 0 | 0.47 |  |
| QoL_MDD_baseline_rTMS | Quality of life of a patient at baseline with MDD | QoL_Baseline_MDD_rTMS | 0.508 | 0 | 0.508 | Study on US patients with rTMS |
| QoL_MDD_nonresponder_pharma | Quality of life patients with MDD - nonresponse to pharma therapy | 0.53 | 0.53 | 0 | 0.53 |  |
| QoL_MDD_remitter_rTMS | Quality of life of a remitter with use of rTMS | QoL_remitter_MDD_rTMS | 0.77 | 0 | 0.77 | Study on US patients with rTMS |
| QoL_MDD_responder_pharma | Quality of life for a MDD responder with drug therapy | QoL_responder_pharma_long_term_followup | 0.69 | 0 | 0.85 |  |
| QoL_MDD_responder_rTMS | QoL responder rTMS under maintenance therapy | QoL_responder_maintenance_therapy_rTMS | 0.663 | 0 | 0.663 |  |
| QoL_stable_condition_post_ECT_or_rTMS | Quality of life for patient in stable condition post treatment with rTMS or ECT | QoL_stable_MDD_post_RX_rTMS_or_ECT | 0.759 | 0 | 0.759 |  |
