# Supplementary material for: Cost effectiveness analysis comparing repetitive transcranial magnetic stimulation to antidepressant medications after a first treatment failure for major depressive disorder in newly diagnosed patients – A lifetime analysis
Source: PLoS One. 2017 Oct 26;12(10):e0186950. doi: 10.1371/journal.pone.0186950 (PMC5658110; doi:10.1371/journal.pone.0186950)
Supplement: S2 Appendix — (DOCX) [file pone.0186950.s023.docx]

S2 Appendix: Distributions

| **DESCRIPTION** | **TYPE** | **PARAMETERS** | **EV** | **COMMENT** |
| --- | --- | --- | --- | --- |
| Quality of life for a responder with drug therapy - 6 month follow up | Triangular | min: 0.67, likeliest: 0.69, max: 0.72 | 0.69333 | Sobocki P, et al. Health-related QoL measured with EQ-5D in patients treated for depression in primary care. Value in Health. 2007. 10(2):153-160 |
| Response rate of rTMS after first failed therapy | Uniform | subtype: 2, low: 0.38, high: 0.56 | 0.47 | Nguyen K-H et al. Cost-effectiveness of repetitive transcranial magnetic stimulation versus antidepressant therapy for treatment-resistant depression. Value in Health. 2015.18:597-604. Based on a second to third response rate of 33.2% to 48.7% in Nguyen and an assumed 15% decrement in the response rate then, a first to second response rate would be: 0.332 * 1.15 = 0.38 to 0.487 * 1.15 = 0.56. Note: This response rate is in line with the 43.5% one year response rate as demonstrated in Philip NS et al. Brain Stimulation 2016;9(20):251-7. |
| Life expectancy of a 55 yr old with MDD | Uniform | subtype: 2, low: 3, high: 29 | 16.0 | Based on life expectancy of MDD derived from: Zivin K et al. Early mortality and years of potential life lost among veterans affairs patients with depression. Psychiat Serv. 2012;63:823-26. Life expectancy of person with MDD is 71.1 +/- 13 years. Therefore for a 55 year old, the range would be: 58-84 or; a lower bound of 3 more years of life to 29 more years of life for a 55 year old. |
| Quality of life stable condition post treatment with either rTMS or ECT | Beta | subtype: 2, alpha: (((0.759)^2)*(1-(0.759))/((0.25)^2)-(0.759)), beta: ((1-(0.759))*(((1-(0.759))*(0.759))/((0.25)^2)-1)) | 0.759 | Vallejo-Torres L. et al. Cost-effectiveness of ECT compared to rTMS for treatment -resistant severe depression: decision model. Psych Med 2015. 45:1459-1470. |
| Number of maintenance sessions per year for rTMS | Triangular | min: 3, likeliest: 4, max: 5 | 4.0 | Nguyen et al. Value in Health 2015; estimated from expert opinion |
| Response rate of rTMS from second failure to third attempt | Uniform | subtype: 2, low: 0.332, high: 0.487 | 0.4095 | Nguyen K-H et al. Cost-effectiveness of repetitive transcranial magnetic stimulation versus antidepressant therapy for treatment-resistant depression. Value in Health. 2015.18:597-604. |
| Probability of remission with ECT treatment after failed first therapy | Triangular | min: 0.2, likeliest: 0.463, max: 0.7 | 0.45433 | Nguyen K-H, et al. Cost effectiveness of repetitive transcranial magnetic stimulation versus antidepressant therapy for treatment- resistant depression. Value in Health. 2015. 18:597-604. |
| Remission rate with second therapy after first failed therapy rTMS | Uniform | subtype: 2, low: 0.236, high: 0.374 | 0.305 | Nguyen K-H et al. Cost-effectiveness of repetitive transcranial magnetic stimulation versus antidepressant therapy for treatment-resistant depression. Value in Health. 2015.18: 597-604. See table 1 page 599 for remission from second to third of 0.197 to 0.312. Each successive treatment is a decrement of 20% in its efficacy Note: Remission rate is: 0.197 * 1.2 = 0.236 (low); 0.312 * 1.2 = 0.374. Note: This remission rate is in line with the 30.4% one year response rate as demonstrated in Philip NS et al. Brain Stimulation 2016;9(20):251-7. |
| Probability of relapse with pharma medications | Uniform | subtype: 2, low: 0.11, high: 0.26 | 0.185 | Primary Care Companion. Jrl Clin Psych 2007. 9(3):214-223. Note: These are similar findings to: Perahia DG et al. Duloxetine in the prevention of relapse of major depressive disorder. Brit Jrl Psychiat 2006. 188:346-353 which showed at relapse rate of 17.4% - 21.9%. |
| Number of treatment sessions rTMS in order to achieve clinical efficacy | Normal | mean: 25.3, stddev: 16.7 | 25.3 | Philip NS, et al. Can medication free, treatment-resistant, depressed patients who initially respond to TMS be maintained off medications? A prospective, 12-month multisite randomized pilot trial. Brain Stimulation. 2016 9(20):251-7. |
| Remission rate with fourth therapy after third failed attempt - rTMS | Uniform | subtype: 2, low: 0.158, high: 0.2496 | 0.2038 | Nguyen K-H et al. Cost-effectiveness of repetitive transcranial magnetic stimulation versus antidepressant therapy for treatment-resistant depression. Value in Health. 2015.18:597-604. 20% decrement in efficacy for each successive treatment. Therefore: 0.197 * 0.8 = 0.158 (low); 0.312 *0.8 = 0.2496 |
| Quality of life remitter with MDD using rTMS | Triangular | min: 0.73, likeliest: 0.77, max: 0.81 | 0.77 | Janicak PG, et al. transcranial magnetic stimulation (TMS) for major depression: a multisite, naturalistic, observational study of quality of life outcomes in clinical practice. CNS Spectrums 2013. 18:322-332. |
| Number of treatment sessions for ECT per treatment cycle | Uniform | subtype: 2, low: 6, high: 12 | 9.0 | Derived from 2016 Aetna coverage policy for ECT |
| Probability of death from MDD per year | Uniform | subtype: 2, low: 0.01, high: 0.03 | 0.02 | Vythilingam M, et al. Psychotic depression and mortality. Amer Jrl Psych 2003. 160:574-576. |
| Response rate of rTMS from third to fourth | Uniform | subtype: 2, low: 0.282, high: 0.414 | 0.348 | Nguyen K-H et al. Cost-effectiveness of repetitive transcranial magnetic stimulation versus antidepressant therapy for treatment-resistant depression. Value in Health. 2015.18:597-604. Based on a decrement in the response rate of 15% for each subsequent treatment then the response rate from third to fourth would be: 0.332 * 0.85 = 0.282 (low) and 0.487 * 0.85 = 0.414 |
| QoL responder under maintenance therapy with rTMS | Normal | mean: 0.663, stddev: 0.32 | 0.663 | Mann R, et al. Putting the "Q" in depression QALYs: a comparison of utility measurement using EQ-5D and SF-6D health related quality of life measures. Soc Psych Psych Epidemiol 2009. 44:569-578. |
| Probability of response with ECT | Beta | subtype: 2, alpha: (((0.3722)^2)*(1-(0.3722))/((0.0521)^2)-(0.3722)), beta: ((1-(0.3722))*(((1-(0.3722))*(0.3722))/((0.0521)^2)-1)) | 0.3722 | Vallejo-Torres L, et al. Cost-effectiveness of electroconvulsive therapy compared to repetitive transcranial magnetic stimulation for treatment-resistant severe depression: a decision model. Psych Med 2015. 45:1459-1470. |
| QoL baseline MDD antidepressants | Triangular | min: 0.44, likeliest: 0.47, max: 0.50 | 0.47 | Sobocki P, et al. Health-related QoL measured with EQ-5D in patients treated for depression in primary care. Value in Health. 2007. 10(2):153-160 |
| Remission rate with third therapy after second failed attempt | Uniform | subtype: 2, low: 0.197, high: 0.312 | 0.2545 | Nguyen K-H et al. Cost-effectiveness of repetitive transcranial magnetic stimulation versus antidepressant therapy for treatment-resistant depression. Value in Health. 2015.18:597-604. See table 1 page 599 of paper for remission rTMS therapy after 2 failed medication therapies. |
| Quality of life for a patient with MDD | Normal | mean: 0.508, stddev: 0.215 | 0.508 | Janicak PG, et al. transcranial magnetic stimulation (TMS) for major depression: a multisite, naturalistic, observational study of quality of life outcomes in clinical practice. CNS Spectrums 2013. 18:322-332. |
