# Supplementary material for: Cost effectiveness analysis comparing repetitive transcranial magnetic stimulation to antidepressant medications after a first treatment failure for major depressive disorder in newly diagnosed patients – A lifetime analysis
Source: PLoS One. 2017 Oct 26;12(10):e0186950. doi: 10.1371/journal.pone.0186950 (PMC5658110; doi:10.1371/journal.pone.0186950)
Supplement: S3 Appendix — (DOCX) [file pone.0186950.s024.docx]

**S3 Appendix – definitions of terms:**

Cost effectiveness analysis: is a form of economic **analysis** that compares the relative **costs** and outcomes (effects such as QALYs) of different courses of action.

Dominance of one therapy versus another: A therapy is said to be dominant compared to another when it both costs less and results in improved health outcomes.

Euro-QoL: Euro-QoL is a standardized instrument for measuring generic health status. The health status measured with Euro-QoL is used for estimating preference weight for that health status, then by combining the weight with time, [quality-adjusted life year (QALY)](https://en.wikipedia.org/wiki/Quality_Adjusted_Life_Years) can be computed.

Incremental Cost Effectiveness Ratio (ICER): The incremental cost-effectiveness ratio (ICER) is a statistic used in cost-effectiveness analysis to summarize the cost-effectiveness of a health care intervention. It is defined by the difference in cost between two possible interventions (numerator), divided by the difference in their effect (denominator). In the example used in the paper, it is the cost of rTMS less the cost of pharmacotherapy (incremental cost) divided by the incremental benefit as identified the total QALYs rTMS less the total QALYs pharmacotherapy. In the United States the ICER threshold is in the neighborhood of $50,000/QALY. In other words, the US health system is willing to pay an additional $50,000 for a new therapy/service/diagnostic for one year of perfect health (QALY = 1).

Markov model: Markov models are commonly used to simulate long-term processes (e.g., an individual’s lifespan). Markov models built in TreeAge Pro often represent discrete-time state transition models (although discrete event modeling is also possible). A discrete-time Markov model usually follows a basic design, such that:

- The time period of interest (i.e., lifetime) is divided into equal intervals, or **cycles** (e.g. years).
- A finite set of mutually exclusive **states** is defined such that, in any given cycle, a member of the cohort is in only one state.
- **Initial probabilities** determine the distribution of cohort members among the possible states at the start of the process (typically the entire cohort starts in the same state).
- A matrix of **transition probabilities**, applied in each successive cycle, defines the possible changes to the state the member is in.
- To calculate an expected value for the model (e.g., net cost and/or quality-adjusted life years [QALYs]), different cost and/or utility **rewards** are accumulated for each interval spent in a particular state.

Monte Carlo simulation: There are situations where it is useful to introduce random, or stochastic, elements into some part of the analysis. The variables and distributions used in the model, allow for randomness to take place as defined by the type of distribution identified for the variable. In such situations, Monte Carlo techniques can be applied and run numerous times, each time varying the unit value in the variable. Summary statistics are then generated. Monte Carlo simulations can provide a measure of how confident one can feel about the findings.

Net Monetary Benefit (NMB): NMBs are another way to express cost-effectiveness. The most cost effective therapy is the one with the highest NMB. The NMB is calculated as follows: Effectiveness (in QALYS) X Willingness to Pay – Costs. In the case of this analysis the willingness to pay was zero (0) so that the NMB value was negative. Since it was negative, the lowest negative number had the highest NMB.

Non-responder: < 50% reduction in symptoms

Quality adjusted life year (QALY): A QALY is a generic measure of the burden of disease, and include both the quality and the quantity of life lived. One QALY equals one year of life of perfect health (i.e. score of “1” on Euro-QoL).

Remission: The near absence of depressive symptoms

Response: A 50% reduction in symptoms

Sensitivity analysis: Deterministic sensitivity analysis can take a variety of forms, including 1-way sensitivity analysis (used in this paper) and tornado diagrams. Sensitivity analysis is used to identify critical uncertainties by examining the extent to which a model’s calculations and recommendations are affected as a consequence of changing selected assumptions/variables (also see tornado diagram).

Tornado diagram: A tornado diagram is a set of one-way sensitivity analyses brought together in a single graph. It can include any number of the variables defined in the tree. In the graph, a horizontal bar is generated for each variable being analyzed. Most commonly, the variables with the greatest effect on the findings – e.g. those in which varying the variable enough will cause a change in decision making on which therapy to use are shown.
