# Supplementary material for: Cost effectiveness analysis comparing repetitive transcranial magnetic stimulation to antidepressant medications after a first treatment failure for major depressive disorder in newly diagnosed patients – A lifetime analysis
Source: PLoS One. 2017 Oct 26;12(10):e0186950. doi: 10.1371/journal.pone.0186950 (PMC5658110; doi:10.1371/journal.pone.0186950)
Supplement: S4 Appendix — (DOCX) [file pone.0186950.s025.docx]

S4 Appendix: rTMS Markov Model - cost and effectiveness by reward state and stage

| **STAGE** | **HEALTH STATE** | **PROBABILITY BEING IN HEALTH STATE DURING STAGE** | **HEALTH STATE COST** | **STAGE COST** | **TOTAL COST (CUM)** | **HEALTH STATE EFF (QALYs)** | **STAGE QALYs** | **CUM QALYs** |
| --- | --- | --- | --- | --- | --- | --- | --- | --- |
| 0 | Treatment | 100.00% | $11,525 | $11,525 | $11,525 | 0.51 | 0.51 | 0.51 |
| 0 | Nonresponders | 0.00% | $0 | $11,525 | $11,525 | 0.00 | 0.51 | 0.51 |
| 0 | Remission/Maintenance Therapy | 0.00% | $0 | $11,525 | $11,525 | 0.00 | 0.51 | 0.51 |
| 0 | Die | 0.00% | $0 | $11,525 | $11,525 | 0.00 | 0.51 | 0.51 |
| 0 | Other treatment - ECT | 0.00% | $0 | $11,525 | $11,525 | 0.00 | 0.51 | 0.51 |
| 1 | Treatment | 34.10% | $3,930 | $11,721 | $23,246 | 0.23 | 0.59 | 1.10 |
| 1 | Nonresponders | 53.00% | $6,108 | $11,721 | $23,246 | 0.27 | 0.59 | 1.10 |
| 1 | Remission/Maintenance Therapy | 12.90% | $1,683 | $11,721 | $23,246 | 0.10 | 0.59 | 1.10 |
| 1 | Die | 0.00% | $0 | $11,721 | $23,246 | 0.00 | 0.59 | 1.10 |
| 1 | Other treatment - ECT | 0.00% | $0 | $11,721 | $23,246 | 0.00 | 0.59 | 1.10 |
| 2 | Treatment | 24.45% | $2,818 | $11,275 | $34,522 | 0.16 | 0.65 | 1.75 |
| 2 | Nonresponders | 35.97% | $4,146 | $11,275 | $34,522 | 0.18 | 0.65 | 1.75 |
| 2 | Remission/Maintenance Therapy | 21.85% | $2,851 | $11,275 | $34,522 | 0.17 | 0.65 | 1.75 |
| 2 | Die | 0.26% | $0 | $11,275 | $34,522 | 0.00 | 0.65 | 1.75 |
| 2 | Other treatment - ECT | 17.47% | $1,461 | $11,275 | $34,522 | 0.13 | 0.65 | 1.75 |
| 3 | Treatment | 17.04% | $1,964 | $11,294 | $45,815 | 0.11 | 0.67 | 2.42 |
| 3 | Nonresponders | 26.39% | $3,042 | $11,294 | $45,815 | 0.13 | 0.67 | 2.42 |
| 3 | Remission/Maintenance Therapy | 34.48% | $4,499 | $11,294 | $45,815 | 0.27 | 0.67 | 2.42 |
| 3 | Die | 0.69% | $0 | $11,294 | $45,815 | 0.00 | 0.67 | 2.42 |
| 3 | Other treatment - ECT | 21.39% | $1,789 | $11,294 | $45,815 | 0.16 | 0.67 | 2.42 |
| 4 | Treatment | 12.20% | $1,406 | $11,412 | $57,227 | 0.08 | 0.69 | 3.11 |
| 4 | Nonresponders | 20.70% | $2,385 | $11,412 | $57,227 | 0.11 | 0.69 | 3.11 |
| 4 | Remission/Maintenance Therapy | 45.35% | $5,918 | $11,412 | $57,227 | 0.35 | 0.69 | 3.11 |
| 4 | Die | 1.38% | $0 | $11,412 | $57,227 | 0.00 | 0.69 | 3.11 |
| 4 | Other treatment - ECT | 20.37% | $1,703 | $11,412 | $57,227 | 0.15 | 0.69 | 3.11 |
| 5 | Treatment | 9.17% | $1,056 | $11,504 | $68,731 | 0.06 | 0.69 | 3.81 |
| 5 | Nonresponders | 17.41% | $2,006 | $11,504 | $68,731 | 0.09 | 0.69 | 3.81 |
| 5 | Remission/Maintenance Therapy | 53.20% | $6,942 | $11,504 | $68,731 | 0.41 | 0.69 | 3.81 |
| 5 | Die | 2.29% | $0 | $11,504 | $68,731 | 0.00 | 0.69 | 3.81 |
| 5 | Other treatment - ECT | 17.94% | $1,500 | $11,504 | $68,731 | 0.14 | 0.69 | 3.81 |
| 6 | Treatment | 7.34% | $846 | $11,535 | $80,266 | 0.05 | 0.69 | 4.50 |
| 6 | Nonresponders | 15.54% | $1,790 | $11,535 | $80,266 | 0.08 | 0.69 | 4.50 |
| 6 | Remission/Maintenance Therapy | 58.25% | $7,600 | $11,535 | $80,266 | 0.45 | 0.69 | 4.50 |
| 6 | Die | 3.36% | $0 | $11,535 | $80,266 | 0.00 | 0.69 | 4.50 |
| 6 | Other treatment - ECT | 15.52% | $1,298 | $11,535 | $80,266 | 0.12 | 0.69 | 4.50 |
| 7 | Treatment | 6.26% | $722 | $11,506 | $91,772 | 0.04 | 0.69 | 5.19 |
| 7 | Nonresponders | 14.47% | $1,668 | $11,506 | $91,772 | 0.07 | 0.69 | 5.19 |
| 7 | Remission/Maintenance Therapy | 61.15% | $7,980 | $11,506 | $91,772 | 0.47 | 0.69 | 5.19 |
| 7 | Die | 4.52% | $0 | $11,506 | $91,772 | 0.00 | 0.69 | 5.19 |
| 7 | Other treatment - ECT | 13.59% | $1,137 | $11,506 | $91,772 | 0.10 | 0.69 | 5.19 |
| 8 | Treatment | 5.64% | $650 | $11,431 | $103,203 | 0.04 | 0.68 | 5.87 |
| 8 | Nonresponders | 13.85% | $1,597 | $11,431 | $103,203 | 0.07 | 0.68 | 5.87 |
| 8 | Remission/Maintenance Therapy | 62.58% | $8,165 | $11,431 | $103,203 | 0.48 | 0.68 | 5.87 |
| 8 | Die | 5.74% | $0 | $11,431 | $103,203 | 0.00 | 0.68 | 5.87 |
| 8 | Other treatment - ECT | 12.19% | $1,019 | $11,431 | $103,203 | 0.09 | 0.68 | 5.87 |
| 9 | Treatment | 5.27% | $608 | $11,324 | $114,527 | 0.03 | 0.67 | 6.55 |
| 9 | Nonresponders | 13.47% | $1,552 | $11,324 | $114,527 | 0.07 | 0.67 | 6.55 |
| 9 | Remission/Maintenance Therapy | 63.04% | $8,226 | $11,324 | $114,527 | 0.49 | 0.67 | 6.55 |
| 9 | Die | 7.00% | $0 | $11,324 | $114,527 | 0.00 | 0.67 | 6.55 |
| 9 | Other treatment - ECT | 11.22% | $938 | $11,324 | $114,527 | 0.09 | 0.67 | 6.55 |
| 10 | Treatment | 5.06% | $583 | $11,198 | $125,725 | 0.03 | 0.67 | 7.21 |
| 10 | Nonresponders | 13.20% | $1,521 | $11,198 | $125,725 | 0.07 | 0.67 | 7.21 |
| 10 | Remission/Maintenance Therapy | 62.92% | $8,211 | $11,198 | $125,725 | 0.48 | 0.67 | 7.21 |
| 10 | Die | 8.26% | $0 | $11,198 | $125,725 | 0.00 | 0.67 | 7.21 |
| 10 | Other treatment - ECT | 10.56% | $883 | $11,198 | $125,725 | 0.08 | 0.67 | 7.21 |
| 11 | Treatment | 4.92% | $567 | $11,060 | $136,785 | 0.03 | 0.66 | 7.87 |
| 11 | Nonresponders | 12.99% | $1,497 | $11,060 | $136,785 | 0.07 | 0.66 | 7.87 |
| 11 | Remission/Maintenance Therapy | 62.46% | $8,150 | $11,060 | $136,785 | 0.48 | 0.66 | 7.87 |
| 11 | Die | 9.51% | $0 | $11,060 | $136,785 | 0.00 | 0.66 | 7.87 |
| 11 | Other treatment - ECT | 10.11% | $846 | $11,060 | $136,785 | 0.08 | 0.66 | 7.87 |
| 12 | Treatment | 4.82% | $556 | $10,916 | $147,701 | 0.03 | 0.65 | 8.52 |
| 12 | Nonresponders | 12.81% | $1,476 | $10,916 | $147,701 | 0.07 | 0.65 | 8.52 |
| 12 | Remission/Maintenance Therapy | 61.81% | $8,065 | $10,916 | $147,701 | 0.48 | 0.65 | 8.52 |
| 12 | Die | 10.76% | $0 | $10,916 | $147,701 | 0.00 | 0.65 | 8.52 |
| 12 | Other treatment - ECT | 9.80% | $820 | $10,916 | $147,701 | 0.07 | 0.65 | 8.52 |
| 13 | Treatment | 4.74% | $547 | $10,770 | $158,471 | 0.03 | 0.64 | 9.15 |
| 13 | Nonresponders | 12.63% | $1,456 | $10,770 | $158,471 | 0.06 | 0.64 | 9.15 |
| 13 | Remission/Maintenance Therapy | 61.06% | $7,967 | $10,770 | $158,471 | 0.47 | 0.64 | 9.15 |
| 13 | Die | 12.00% | $0 | $10,770 | $158,471 | 0.00 | 0.64 | 9.15 |
| 13 | Other treatment - ECT | 9.57% | $800 | $10,770 | $158,471 | 0.07 | 0.64 | 9.15 |
| 14 | Treatment | 4.67% | $539 | $10,622 | $169,093 | 0.03 | 0.63 | 9.78 |
| 14 | Nonresponders | 12.46% | $1,436 | $10,622 | $169,093 | 0.06 | 0.63 | 9.78 |
| 14 | Remission/Maintenance Therapy | 60.26% | $7,863 | $10,622 | $169,093 | 0.46 | 0.63 | 9.78 |
| 14 | Die | 13.22% | $0 | $10,622 | $169,093 | 0.00 | 0.63 | 9.78 |
| 14 | Other treatment - ECT | 9.39% | $785 | $10,622 | $169,093 | 0.07 | 0.63 | 9.78 |
| 15 | Treatment | 4.61% | $531 | $10,476 | $179,569 | 0.03 | 0.62 | 10.40 |
| 15 | Nonresponders | 12.29% | $1,417 | $10,476 | $179,569 | 0.06 | 0.62 | 10.40 |
| 15 | Remission/Maintenance Therapy | 59.44% | $7,756 | $10,476 | $179,569 | 0.46 | 0.62 | 10.40 |
| 15 | Die | 14.43% | $0 | $10,476 | $179,569 | 0.00 | 0.62 | 10.40 |
| 15 | Other treatment - ECT | 9.23% | $772 | $10,476 | $179,569 | 0.07 | 0.62 | 10.40 |
| 16 | Treatment | 4.55% | $524 | $10,331 | $189,900 | 0.03 | 0.61 | 11.02 |
| 16 | Nonresponders | 12.13% | $1,398 | $10,331 | $189,900 | 0.06 | 0.61 | 11.02 |
| 16 | Remission/Maintenance Therapy | 58.62% | $7,649 | $10,331 | $189,900 | 0.45 | 0.61 | 11.02 |
| 16 | Die | 15.62% | $0 | $10,331 | $189,900 | 0.00 | 0.61 | 11.02 |
| 16 | Other treatment - ECT | 9.09% | $760 | $10,331 | $189,900 | 0.07 | 0.61 | 11.02 |
