# Supplementary material for: Cost effectiveness analysis comparing repetitive transcranial magnetic stimulation to antidepressant medications after a first treatment failure for major depressive disorder in newly diagnosed patients – A lifetime analysis
Source: PLoS One. 2017 Oct 26;12(10):e0186950. doi: 10.1371/journal.pone.0186950 (PMC5658110; doi:10.1371/journal.pone.0186950)
Supplement: S5 Appendix — (DOCX) [file pone.0186950.s026.docx]

S5 Appendix: Medication Markov Model - cost and effectiveness by reward state and stage

| **STAGE** | **HEALTH STATE** | **PROBABILITY BEING IN HEALTH STATE DURING STAGE** | **HEALTH STATE COST** | **STAGE COST** | **TOTAL COST (CUM)** | **HEALTH STATE EFF (QALYs)** | **STAGE QALYs** | **CUM QALYs** |
| --- | --- | --- | --- | --- | --- | --- | --- | --- |
| 0 | Treatment | 100.00% | $13,048 | $13,048 | $13,048 | 0.47 | 0.47 | 0.47 |
| 0 | Nonresponders | 0.00% | $0 | $13,048 | $13,048 | 0.00 | 0.47 | 0.47 |
| 0 | Die | 0.00% | $0 | $13,048 | $13,048 | 0.00 | 0.47 | 0.47 |
| 0 | Other treatment - ECT | 0.00% | $0 | $13,048 | $13,048 | 0.00 | 0.47 | 0.47 |
| 0 | Remission/Maintenance therapy | 0.00% | $0 | $13,048 | $13,048 | 0.00 | 0.47 | 0.47 |
| 1 | Treatment | 3.25% | $424 | $13,048 | $26,097 | 0.02 | 0.55 | 1.02 |
| 1 | Nonresponders | 87.00% | $11,352 | $13,048 | $26,097 | 0.46 | 0.55 | 1.02 |
| 1 | Die | 0.00% | $0 | $13,048 | $26,097 | 0.00 | 0.55 | 1.02 |
| 1 | Other treatment - ECT | 0.00% | $0 | $13,048 | $26,097 | 0.00 | 0.55 | 1.02 |
| 1 | Remission/Maintenance therapy | 9.75% | $1,272 | $13,048 | $26,097 | 0.07 | 0.55 | 1.02 |
| 2 | Treatment | 4.58% | $598 | $13,023 | $39,120 | 0.03 | 0.67 | 1.70 |
| 2 | Nonresponders | 31.91% | $4,163 | $13,023 | $39,120 | 0.17 | 0.67 | 1.70 |
| 2 | Die | 0.19% | $0 | $13,023 | $39,120 | 0.00 | 0.67 | 1.70 |
| 2 | Other treatment - ECT | 53.09% | $6,927 | $13,023 | $39,120 | 0.40 | 0.67 | 1.70 |
| 2 | Remission/Maintenance therapy | 10.23% | $1,335 | $13,023 | $39,120 | 0.07 | 0.67 | 1.70 |
| 3 | Treatment | 1.79% | $234 | $12,996 | $52,116 | 0.01 | 0.70 | 2.39 |
| 3 | Nonresponders | 15.85% | $2,069 | $12,996 | $52,116 | 0.08 | 0.70 | 2.39 |
| 3 | Die | 0.40% | $0 | $12,996 | $52,116 | 0.00 | 0.70 | 2.39 |
| 3 | Other treatment - ECT | 48.44% | $6,321 | $12,996 | $52,116 | 0.37 | 0.70 | 2.39 |
| 3 | Remission/Maintenance therapy | 33.52% | $4,373 | $12,996 | $52,116 | 0.23 | 0.70 | 2.39 |
| 4 | Treatment | 0.87% | $114 | $12,909 | $65,025 | 0.01 | 0.69 | 3.08 |
| 4 | Nonresponders | 12.61% | $1,645 | $12,909 | $65,025 | 0.07 | 0.69 | 3.08 |
| 4 | Die | 1.07% | $0 | $12,909 | $65,025 | 0.00 | 0.69 | 3.08 |
| 4 | Other treatment - ECT | 36.11% | $4,711 | $12,909 | $65,025 | 0.27 | 0.69 | 3.08 |
| 4 | Remission/Maintenance therapy | 49.34% | $6,438 | $12,909 | $65,025 | 0.34 | 0.69 | 3.08 |
| 5 | Treatment | 0.68% | $88 | $12,780 | $77,805 | 0.00 | 0.67 | 3.76 |
| 5 | Nonresponders | 13.66% | $1,783 | $12,780 | $77,805 | 0.07 | 0.67 | 3.76 |
| 5 | Die | 2.06% | $0 | $12,780 | $77,805 | 0.00 | 0.67 | 3.76 |
| 5 | Other treatment - ECT | 27.40% | $3,575 | $12,780 | $77,805 | 0.21 | 0.67 | 3.76 |
| 5 | Remission/Maintenance therapy | 56.21% | $7,334 | $12,780 | $77,805 | 0.39 | 0.67 | 3.76 |
| 6 | Treatment | 0.72% | $95 | $12,633 | $90,439 | 0.01 | 0.66 | 4.42 |
| 6 | Nonresponders | 15.07% | $1,966 | $12,633 | $90,439 | 0.08 | 0.66 | 4.42 |
| 6 | Die | 3.18% | $0 | $12,633 | $90,439 | 0.00 | 0.66 | 4.42 |
| 6 | Other treatment - ECT | 23.29% | $3,039 | $12,633 | $90,439 | 0.18 | 0.66 | 4.42 |
| 6 | Remission/Maintenance therapy | 57.74% | $7,534 | $12,633 | $90,439 | 0.40 | 0.66 | 4.42 |
| 7 | Treatment | 0.80% | $104 | $12,483 | $102,922 | 0.01 | 0.65 | 5.07 |
| 7 | Nonresponders | 15.83% | $2,065 | $12,483 | $102,922 | 0.08 | 0.65 | 5.07 |
| 7 | Die | 4.34% | $0 | $12,483 | $102,922 | 0.00 | 0.65 | 5.07 |
| 7 | Other treatment - ECT | 21.90% | $2,858 | $12,483 | $102,922 | 0.17 | 0.65 | 5.07 |
| 7 | Remission/Maintenance therapy | 57.14% | $7,455 | $12,483 | $102,922 | 0.40 | 0.65 | 5.07 |
| 8 | Treatment | 0.84% | $110 | $12,334 | $115,255 | 0.01 | 0.64 | 5.71 |
| 8 | Nonresponders | 16.02% | $2,091 | $12,334 | $115,255 | 0.08 | 0.64 | 5.71 |
| 8 | Die | 5.48% | $0 | $12,334 | $115,255 | 0.00 | 0.64 | 5.71 |
| 8 | Other treatment - ECT | 21.61% | $2,820 | $12,334 | $115,255 | 0.16 | 0.64 | 5.71 |
| 8 | Remission/Maintenance therapy | 56.05% | $7,314 | $12,334 | $115,255 | 0.39 | 0.64 | 5.71 |
| 9 | Treatment | 0.85% | $111 | $12,187 | $127,443 | 0.01 | 0.64 | 6.35 |
| 9 | Nonresponders | 15.92% | $2,078 | $12,187 | $127,443 | 0.08 | 0.64 | 6.35 |
| 9 | Die | 6.60% | $0 | $12,187 | $127,443 | 0.00 | 0.64 | 6.35 |
| 9 | Other treatment - ECT | 21.57% | $2,814 | $12,187 | $127,443 | 0.16 | 0.64 | 6.35 |
| 9 | Remission/Maintenance therapy | 55.06% | $7,184 | $12,187 | $127,443 | 0.38 | 0.64 | 6.35 |
| 10 | Treatment | 0.85% | $110 | $12,044 | $139,486 | 0.01 | 0.63 | 6.98 |
| 10 | Nonresponders | 15.72% | $2,051 | $12,044 | $139,486 | 0.08 | 0.63 | 6.98 |
| 10 | Die | 7.70% | $0 | $12,044 | $139,486 | 0.00 | 0.63 | 6.98 |
| 10 | Other treatment - ECT | 21.49% | $2,804 | $12,044 | $139,486 | 0.16 | 0.63 | 6.98 |
| 10 | Remission/Maintenance therapy | 54.25% | $7,078 | $12,044 | $139,486 | 0.38 | 0.63 | 6.98 |
| 11 | Treatment | 0.84% | $109 | $11,902 | $151,388 | 0.01 | 0.62 | 7.60 |
| 11 | Nonresponders | 15.51% | $2,023 | $11,902 | $151,388 | 0.08 | 0.62 | 7.60 |
| 11 | Die | 8.79% | $0 | $11,902 | $151,388 | 0.00 | 0.62 | 7.60 |
| 11 | Other treatment - ECT | 21.32% | $2,782 | $11,902 | $151,388 | 0.16 | 0.62 | 7.60 |
| 11 | Remission/Maintenance therapy | 53.56% | $6,988 | $11,902 | $151,388 | 0.37 | 0.62 | 7.60 |
| 12 | Treatment | 0.82% | $108 | $11,762 | $163,151 | 0.01 | 0.61 | 8.21 |
| 12 | Nonresponders | 15.30% | $1,997 | $11,762 | $163,151 | 0.08 | 0.61 | 8.21 |
| 12 | Die | 9.86% | $0 | $11,762 | $163,151 | 0.00 | 0.61 | 8.21 |
| 12 | Other treatment - ECT | 21.09% | $2,752 | $11,762 | $163,151 | 0.16 | 0.61 | 8.21 |
| 12 | Remission/Maintenance therapy | 52.92% | $6,905 | $11,762 | $163,151 | 0.37 | 0.61 | 8.21 |
| 13 | Treatment | 0.81% | $106 | $11,624 | $174,775 | 0.01 | 0.61 | 8.82 |
| 13 | Nonresponders | 15.12% | $1,972 | $11,624 | $174,775 | 0.08 | 0.61 | 8.82 |
| 13 | Die | 10.91% | $0 | $11,624 | $174,775 | 0.00 | 0.61 | 8.82 |
| 13 | Other treatment - ECT | 20.85% | $2,721 | $11,624 | $174,775 | 0.16 | 0.61 | 8.82 |
| 13 | Remission/Maintenance therapy | 52.31% | $6,825 | $11,624 | $174,775 | 0.36 | 0.61 | 8.82 |
| 14 | Treatment | 0.80% | $105 | $11,488 | $186,263 | 0.01 | 0.60 | 9.42 |
| 14 | Nonresponders | 14.94% | $1,949 | $11,488 | $186,263 | 0.08 | 0.60 | 9.42 |
| 14 | Die | 11.96% | $0 | $11,488 | $186,263 | 0.00 | 0.60 | 9.42 |
| 14 | Other treatment - ECT | 20.60% | $2,688 | $11,488 | $186,263 | 0.16 | 0.60 | 9.42 |
| 14 | Remission/Maintenance therapy | 51.70% | $6,746 | $11,488 | $186,263 | 0.36 | 0.60 | 9.42 |
| 15 | Treatment | 0.79% | $104 | $11,353 | $197,616 | 0.01 | 0.59 | 10.01 |
| 15 | Nonresponders | 14.76% | $1,926 | $11,353 | $197,616 | 0.08 | 0.59 | 10.01 |
| 15 | Die | 13.00% | $0 | $11,353 | $197,616 | 0.00 | 0.59 | 10.01 |
| 15 | Other treatment - ECT | 20.36% | $2,656 | $11,353 | $197,616 | 0.15 | 0.59 | 10.01 |
| 15 | Remission/Maintenance therapy | 51.09% | $6,667 | $11,353 | $197,616 | 0.35 | 0.59 | 10.01 |
| 16 | Treatment | 0.78% | $0 | $11,117 | $208,733 | 0.01 | 0.59 | 10.60 |
| 16 | Nonresponders | 14.59% | $1,903 | $11,117 | $208,733 | 0.08 | 0.59 | 10.60 |
| 16 | Die | 14.02% | $0 | $11,117 | $208,733 | 0.00 | 0.59 | 10.60 |
| 16 | Other treatment - ECT | 20.11% | $2,625 | $11,117 | $208,733 | 0.15 | 0.59 | 10.60 |
| 16 | Remission/Maintenance therapy | 50.50% | $6,589 | $11,117 | $208,733 | 0.35 | 0.59 | 10.60 |
