# Supplementary material for: Cost effectiveness analysis comparing repetitive transcranial magnetic stimulation to antidepressant medications after a first treatment failure for major depressive disorder in newly diagnosed patients – A lifetime analysis
Source: PLoS One. 2017 Oct 26;12(10):e0186950. doi: 10.1371/journal.pone.0186950 (PMC5658110; doi:10.1371/journal.pone.0186950)
Supplement: S6 Appendix — (DOCX) [file pone.0186950.s027.docx]

S6 Appendix: Equations used in Markov Model

| ***rTMS*** |  | |  | |
| --- | --- | --- | --- | --- |
| **Treatment rTMS** |  | |  | |
| Rewards | Duration | Values | |  |
| Initial cost | First year | CPT_90867+CPT_90868*Number_rTMS_sessions_treatment+CPT_90869+Cost_Medical_Services_yearly+CPT_99214*2+CPT_90832*52 | |  |
| Incremental cost | Intervening yrs | CPT_90867+CPT_90868*Number_rTMS_sessions_treatment+CPT_90869+Cost_Medical_Services_yearly+CPT_99214*2+CPT_90832*52 | |  |
| Final cost | Final year | CPT_90867+CPT_90868*Number_rTMS_sessions_treatment+CPT_90869+Cost_Medical_Services_yearly+CPT_99214*2+CPT_90832*52 | |  |
| Initial effectiveness | First year | QoL_MDD_baseline_rTMS | |  |
| Incr. effectiveness | Intervening yrs | QoL_MDD_responder_rTMS | |  |
| Final effectiveness | Final year | QoL_MDD_responder_rTMS | |  |
|  |  |  | |  |
| **Non-responder rTMS** |  |  | |  |
| Rewards | Duration | Values | |  |
| Initial cost | First year | CPT_90867+CPT_90868*Number_rTMS_sessions_treatment+CPT_90869+Cost_Medical_Services_yearly+CPT_99214*2+CPT_90832*52 | |  |
| Incremental cost | Intervening yrs | CPT_90867+CPT_90868*Number_rTMS_sessions_treatment+CPT_90869+Cost_Medical_Services_yearly+CPT_99214*2+CPT_90832*52 | |  |
| Final cost | Final year | CPT_90867+CPT_90868*Number_rTMS_sessions_treatment+CPT_90869+Cost_Medical_Services_yearly+CPT_99214*2+CPT_90832*52 | |  |
| Initial effectiveness | First year | QoL_MDD_baseline_rTMS | |  |
| Incr. effectiveness | Intervening yrs | QoL_MDD_baseline_rTMS | |  |
| Final effectiveness | Final year | QoL_MDD_baseline_rTMS | |  |
|  |  |  | |  |
| **Remission/Maintenance Therapy rTMS** |  |  | |  |
| Rewards | Duration | Values | |  |
| Initial cost | First year | CPT_90867+CPT_90868*Number_rTMS_sessions_maintenance+CPT_90869+CPT_99214*2 | |  |
| Incremental cost | Intervening yrs | Cost_antidepressant_meds*12+Cost_Medical_Services_yearly+CPT_99214*4+CPT_90832*52 | |  |
| Final cost | Final year | Cost_antidepressant_meds*12+Cost_Medical_Services_yearly+CPT_99214*4+CPT_90832*52 | |  |
| Initial effectiveness | First year | QoL_MDD_remitter_rTMS | |  |
| Incr. effectiveness | Intervening yrs | QoL_MDD_remitter_rTMS | |  |
| Final effectiveness | Final year | QoL_MDD_remitter_rTMS | |  |
|  |  |  | |  |
| **ECT** |  |  | |  |
| Rewards | Duration | Values | |  |
| Initial cost | First year | (APC_5723+CPT_00104+CPT_90870)*Number_ECT_sessions | |  |
| Incremental cost | Intervening yrs | Cost_Medical_Services_yearly+CPT_99214*2+CPT_90832*52 | |  |
| Final cost | Final year | Cost_Medical_Services_yearly+CPT_99214*2+CPT_90832*52 | |  |
| Initial effectiveness | First year | QoL_MDD_baseline_rTMS | |  |
| Incr. effectiveness | Intervening yrs | QoL_stable_condition_post_ECT_or_rTMS | |  |
| Final effectiveness | Final year | QoL_stable_condition_post_ECT_or_rTMS | |  |
|  |  |  | |  |
| **Death** |  |  | |  |
| Rewards | Duration | Values | |  |
| Initial cost | First year | 0 | |  |
| Incremental cost | Intervening yrs | 0 | |  |
| Final cost | Final year | 0 | |  |
| Initial effectiveness | First year | 0 | |  |
| Incr. effectiveness | Intervening yrs | 0 | |  |
| Final effectiveness | Final year | 0 | |  |
|  |  |  | |  |
| ***Pharmacologic therapy*** |  |  | |  |
| **Treatment Pharma** |  |  | |  |
| Rewards | Duration | Values | |  |
| Initial cost | First year | Cost_antidepressant_meds*12+Cost_Medical_Services_yearly+CPT_99214*4+CPT_90832*52 | |  |
| Incremental cost | Intervening yrs | Cost_antidepressant_meds*12+Cost_Medical_Services_yearly+CPT_99214*4+CPT_90832*52 | |  |
| Final cost | Final year | Cost_antidepressant_meds*12+Cost_Medical_Services_yearly+CPT_99214*4+CPT_90832*52 | |  |
| Initial effectiveness | First year | QoL_MDD_baseline_drug_therapy | |  |
| Incr. effectiveness | Intervening yrs | QoL_MDD_responder_pharma | |  |
| Final effectiveness | Final year | QoL_MDD_responder_pharma | |  |
|  |  |  | |  |
| **Non-responders Pharma** |  |  | |  |
| Rewards | Duration | Values | |  |
| Initial cost | First year | Cost_antidepressant_meds*12+Cost_Medical_Services_yearly+CPT_99214*4+CPT_90832*52 | |  |
| Incremental cost | Intervening yrs | Cost_antidepressant_meds*12+Cost_Medical_Services_yearly+CPT_99214*4+CPT_90832*52 | |  |
| Final cost | Final year | Cost_antidepressant_meds*12+Cost_Medical_Services_yearly+CPT_99214*4+CPT_90832*52 | |  |
| Initial effectiveness | First year | QoL_MDD_nonresponder_pharma | |  |
| Incr. effectiveness | Intervening yrs | QoL_MDD_nonresponder_pharma | |  |
| Final effectiveness | Final year | QoL_MDD_nonresponder_pharma | |  |
|  |  |  | |  |
| **Remission/Maintenance Therapy Pharma** |  |  | |  |
| Rewards | Duration | Values | |  |
| Initial cost | First year | Cost_antidepressant_meds*12+Cost_Medical_Services_yearly+CPT_99214*4+CPT_90832*52 | |  |
| Incremental cost | Intervening yrs | Cost_antidepressant_meds*12+Cost_Medical_Services_yearly+CPT_99214*4+CPT_90832*52 | |  |
| Final cost | Final year | Cost_antidepressant_meds*12+Cost_Medical_Services_yearly+CPT_99214*4+CPT_90832*52 | |  |
| Initial effectiveness | First year | QoL_MDD_responder_pharma | |  |
| Incr. effectiveness | Intervening yrs | QoL_MDD_responder_pharma | |  |
| Final effectiveness | Final year | QoL_MDD_responder_pharma | |  |
|  |  |  | |  |
| **ECT** |  |  | |  |
| Rewards | Duration | Values | |  |
| Initial cost | First year | (APC_5723+CPT_00104+CPT_90870)*Number_ECT_sessions | |  |
| Incremental cost | Intervening yrs | Cost_antidepressant_meds*12+Cost_Medical_Services_yearly+CPT_99214*4+CPT_90832*52 | |  |
| Final cost | Final year | Cost_antidepressant_meds*12+Cost_Medical_Services_yearly+CPT_99214*4+CPT_90832*52 | |  |
| Initial effectiveness | First year | QoL_MDD_baseline_drug_therapy | |  |
| Incr. effectiveness | Intervening yrs | QoL_stable_condition_post_ECT_or_rTMS | |  |
| Final effectiveness | Final year | QoL_stable_condition_post_ECT_or_rTMS | |  |
|  |  |  | |  |
| **Death** |  |  | |  |
| Rewards | Duration | Values | |  |
| Initial cost | First year | 0 | |  |
| Incremental cost | Intervening yrs | 0 | |  |
| Final cost | Final year | 0 | |  |
| Initial effectiveness | First year | 0 | |  |
| Incr. effectiveness | Intervening yrs | 0 | |  |
| Final effectiveness | Final year | 0 | |  |
